# Associations between anxiety and working memory components in clinically evaluated children with and without ADHD

**DOI:** 10.3389/fpsyt.2025.1536942

**Published:** 2025-06-13

**Authors:** Carolyn L. Marsh, Fatou Gaye, Enrique Cibrian, Sooyun Cho, Miho O. Tatsuki, Julia O. Obi, Meaghan E. Geren, Sherelle L. Harmon, Michael J. Kofler

**Affiliations:** Department of Psychology, Florida State University, Tallahassee, FL, United States

**Keywords:** working memory, anxiety, ADHD, worry, anxious arousal

## Abstract

Theoretical models describe working memory difficulties as risk factors and/or outcomes of anxiety in children, but the current evidence base is surprisingly mixed. Understanding the nature of the working memory/anxiety relation is complicated by the multi-component nature of each of these constructs. Consideration of the co-occurrence of anxiety with attention-deficit/hyperactivity disorder (ADHD) is also imperative given that ADHD is associated with large magnitude working memory impairments. The current study addressed these considerations using bifactor modeling to evaluate associations between latent estimates of working memory and anxiety subcomponents. The carefully-phenotyped sample included *N*=340 children between the ages of 8 and 13 (*M* = 10.31, *SD* = 1.39; 144 female participants), with an oversampling of children with ADHD (*n*=197). Results showed that domain-general anxiety was associated with worse phonological short-term memory (*r* = -.22, *p* = .01), but not central executive working memory or visuospatial short-term memory. Domain-specific anxiety factors (cognitive worry, physiological arousal) did not uniquely predict any of the short-term/working memory components. Further, multigroup analysis indicated that the magnitude and significance of these relations were comparable for both children with and without ADHD. Our findings did not support unique relations between domain-specific cognitive worry/physiological arousal and instead implicated domain-general common anxiety in difficulties with phonological short-term memory. Further research will be needed to replicate findings using this approach across additional measures and performance metrics, while continuing to account for the high co-occurrence between anxiety and ADHD.

## Introduction

Working memory is an executive function that involves the active, top-down manipulation of information held in short-term memory through interrelated functions of updating, dual-processing, and temporal/serial reordering (1). Working memory is crucial to development and has been associated with a wide range of behavioral and functional outcomes, such as academic (2), social (3), and occupational (4) functioning. Further, working memory has been found to be associated with various forms of psychopathology in children (5), including anxiety, a highly prevalent form of internalizing problems in childhood characterized by a variety of symptoms including worry, fear, avoidance, vigilance, and hyperarousal (6–8). As a result, understanding the nature of, and the processes underlying, working memory’s relation to anxiety has the potential to provide essential insights into the interplay between neurocognitive, behavioral, and emotional functioning in pediatric populations. However, characterizing the relations between working memory and anxiety is complicated given the multi-component structure of working memory (9) and multidimensional nature of anxiety (10). Additionally, anxiety frequently co-occurs with attention-deficit/hyperactivity disorder (ADHD; 11), a neurodevelopmental disorder characterized by inattention and hyperactivity/impulsivity (6) that has been linked with large magnitude impairments in working memory (12, 13). As a result, co-occurring ADHD is also an important variable to consider when evaluating these working memory and anxiety relations. Building on prior work, the current study is the first to fractionate the working memory system into its component processes (i.e., central executive, phonological short-term memory, visuospatial short-term memory; defined below) and examine their relations with theoretically motivated dimensions of anxiety (i.e., cognitive worry, physiological arousal) using a latent variable approach. These relations will also be examined while accounting for ADHD in a large and well-characterized sample of clinically evaluated children.

### Anxiety and working memory

Working memory deficits have been proposed to be an outcome of (14), risk factor for (7), and/or reciprocally related to (15, 16) anxiety symptoms. In general, the mechanisms by which anxiety may be related to impaired working memory are theorized to be a combination of top-down and bottom-up cognitive processes (15). At the bottom-up level, greater anxiety is related to worse filtering efficiency (16, 17) due to prioritization of threat-related cues (e.g., worry thoughts or external stimuli; 14, 16). From a top-down perspective, there is competition for cognitive resources and interference between anxiety-related and task-related processes (16). This competition is evidenced by similar neural circuitry involved in both working memory and anxiety (18), which reduces bandwidth for both storing and processing task-relevant information (1, 14). In a reciprocal fashion, depleted attentional control resources then make it difficult to disengage from cognitive processes of anxiety (i.e., worry thoughts), which subsequently results in increased dual-processing working memory demands (1, 15, 19). However, others have argued that anxiety may also serve a motivational function that potentially offsets the negative effects of anxiety on working memory (20). Theoretical work suggests that increased motivation results in individuals compensating for impaired attentional control through greater recruitment of cognitive resources and increased effort (14, 21).

Prior work examining working memory and anxiety in children and adolescents has yielded mixed results. Some studies have found increased anxiety to be related to worse working memory (16, 22–24), and others have found no effect (25) or even the opposite relation (26–28). The mixed literature is highlighted in varying meta-analytic studies with several methodological differences such as examining anxiety dimensionally (i.e., continuum of severity) versus categorically (i.e., diagnostic categories). One meta-analysis found a small overall relation between greater dimensional levels of anxiety and reduced working memory capacity (*d* = -0.33; 16), whereas another found no relation (25). When examining anxiety categorically, the most recent meta-analysis found a small effect in the opposite direction such that better working memory accuracy was found among anxiety disorder groups compared to control groups (*d* = 0.38; 27), a finding that was also demonstrated in a recent empirical study controlling for ADHD status (28). Taken together, relations between anxiety and working memory are likely impacted by multiple factors, such as symptom severity and clinical significance, as well as the multicomponent nature of both anxiety and working memory.

A primary limitation that may contribute to these disparities is that previous studies have typically examined relations based on a single measure of working memory. Use of a single task significantly limits our ability to infer construct-level associations (16) because the majority of variance in any single neurocognitive test is attributable to process(es) other than the specific executive function of interest (29). Additionally, a large body of evidence indicates that working memory is not a unitary construct (for review, see 30). An influential framework of working memory with significant empirical support proposed by Baddeley (9) suggests that working memory may be broken down into three components. First, the *central executive* is responsible for operating on the information stored in short-term memory, hence the “working” part of working memory. Central executive processes include reordering and updating stimuli held in short-term memory, as well as maintaining relevant information in the forefront of memory while performing a secondary, cognitively demanding task (i.e., dual-processing) (1). In addition to the central executive, Baddeley (9) proposed two temporary storage and rehearsal, or short-term memory, systems: the visuospatial sketchpad (visuospatial short-term memory) and the phonological loop (phonological short-term memory). The *visuospatial short-term memory* component is responsible for visual and spatial information, whereas the *phonological short-term memory* component is responsible for language-based verbal information. These three components of working memory are both functionally and anatomically distinct (9, 31). However, single tests cannot measure just one working memory component because the central executive requires information to operate on (i.e., information from phonological and visuospatial short-term memory systems), and at least some central executive processes are evoked even by simple span/short-term memory tasks (9, 29). An additional short-term storage component, the episodic buffer, was added to the model more recently to account for bound, cross-modality information (32). The episodic buffer was not investigated in the current study in order to examine modality-specific processes but will be an important component to consider in future studies.

### Dimensions of anxiety and components of working memory

Despite extensive research on overall relations between anxiety and working memory, the specific processes and systems that might be driving or masking these relations cannot be determined because, to our knowledge, no studies have examined the multidimensional/multi-component relations between anxiety and working memory components at a latent variable level (16). However, theoretical models generally posit that difficulties in the domain-general central executive are driving the hypothesized working memory/anxiety relations (14, 16) due to anxiety negatively affecting attentional control (33). Specifically, Moran (16) found similar magnitude relations between anxiety and performance on visuospatial (*d* = -0.41) and phonological (*d* = -0.34) working memory tasks in a large meta-analysis of both adult and child samples. Based on this similarity, Moran (16) posited that overall anxiety was likely to be associated with the variance shared between visuospatial and phonological working memory tasks (i.e., domain-general central executive).

In contrast, others have argued that the short-term memory stores are implicated in specific, separable dimensions of anxiety (20, 34): physiological arousal and cognitive worry (35–37). *Physiological arousal* (i.e., anxious arousal) refers to somatic symptoms and arousal such as hypervigilance, increased heart rate, sweating, dizziness, and somatic tension (35). *Cognitive worry* (i.e., anxious apprehension) on the other hand refers to worry and rumination about negative events that may happen in the future (35). Some evidence suggests that arousal may be uniquely associated with visuospatial memory tasks and worry may be uniquely associated with phonological memory tasks (20, 38). The importance of examining these two dimensions of anxiety and their relations with working memory has been highlighted (39). For example, evidence suggests that physiological arousal and engagement with visuospatial memory tasks involve similar right prefrontal and right posterior parietal brain regions (i.e., asymmetric dependency). It is hypothesized these shared brain regions result in disruption of visuospatial short-term memory processes due to competition for limited neural resources (20, 34). Similarly, worry and engagement with phonological memory tasks both involve regions in the prefrontal cortex and left-hemisphere verbal processing circuits that may lead to competition for cognitive resources (20, 36). Notably however, worry may be more readily regulated than arousal by top-down processes when needed to meet the demands of high cognitive load tasks (20, 40). Despite overlap in reliance on certain prefrontal systems between physiological arousal/visuospatial tasks and cognitive worry/phonological tasks, there is also evidence for engagement of separable shared systems between the two pairs (20, 41). Although these studies reflect methodological refinements including differentiating between anxiety dimensions and assessing multiple working memory modalities, no studies have fractionated performance on multiple working memory tests into the central executive, and visuospatial and phonological short-term memory subsystems.

Based on this evidence, Moran (16) proposed a model in which greater anxiety is related to impairments in each of the three working memory components. Specifically, the model proposes that common anxiety (i.e., shared variance between arousal and worry) predicts domain-general attentional control (i.e., central executive working memory), whereas variance specific to worry and arousal predict phonological and visuospatial short-term storage capacity, respectively. Importantly, however, Moran (16) called for studies to examine these hypotheses within the same study because the conclusions were based on inferences from comparisons across different studies. Given the emphasis on distinct dimensions of anxiety in the literature, the shared variance between arousal and worry (common anxiety) is not clearly defined, but could represent temperamental characteristics, attentional biases, or aspects of emotion regulation and cognitive control that are shared between these constructs (42–44). The Moran (16) review emphasized the need for a latent-variable approach to isolate unique variance associated with each construct of this model, a method that had not been used in studies prior to their meta-analysis or since then, to our knowledge. Indeed, Gustavson and Miyake (39) highlighted the importance of taking the multifaceted nature of both working memory and anxiety into account when investigating and characterizing the relation between working memory and anxiety. This is the approach taken in the current study.

### Co-occurring anxiety and ADHD

In addition to the need for increased specificity in examining relations between the subcomponents of both working memory and anxiety, accounting for the role of co-occurring psychopathology is also important (27). In particular, anxiety commonly co-occurs with ADHD (11), as approximately 25% of children with ADHD have co-occurring anxiety and vice versa (11, 45). The comorbidity of anxiety and ADHD presents a critical consideration for understanding relations between anxiety and working memory given that working memory difficulties are very common in ADHD (31). Estimates suggest that the majority of children with ADHD have a deficit in this area (i.e., 65-85%; 12, 13, 31, 46).

Research using latent variable methods suggests that working memory impairments in ADHD are largely driven by deficits in the central executive, rather than the two short-term memory storage systems (1, 31). For example, Kofler et al. (31) found that the central executive, but neither of the short-term memory systems, was uniquely associated with ADHD symptom severity. However, although short-term memory deficits do not appear to underlie ADHD symptomology (31), there is nonetheless evidence that children with ADHD demonstrate greater visuospatial than phonological short-term memory impairments. Findings further suggest that phonological short-term memory tends to be intact in most children with ADHD (31, 47, 48). Overall, experimental and theoretical work has implicated central executive working memory deficits as a causal factor in ADHD symptom expression (e.g., 49, 50). Thus, examining the role of co-occurring ADHD in the relations between working memory and anxiety is critical, particularly when examining these associations in pediatric populations.

Interestingly, theoretical work predicts that anxiety is related to working memory impairment above and beyond what can be accounted for by ADHD (21, 22, 51). However, empirical studies that have specifically taken ADHD diagnostic status or symptoms into account when evaluating the relations between anxiety and working memory in youth have yielded highly mixed results (22, 28, 52–54). Importantly, however, none of these studies were able to consider the multi-component nature of working memory or the multiple dimensions of anxiety (16). In addition, to our knowledge, the majority of these studies used tests that have been criticized for poor construct validity and are likely better tests of short-term memory and/or gross neuropsychological functioning rather than working memory (for reviews, see 29, 30, 55). Given that the ADHD and anxiety literatures have both emphasized the importance of these methodological considerations, addressing these limitations is crucial to advancing our understanding of the associations between the multidimensional/multi-component anxiety and working memory constructs. Further, the extent to which these hypothesized anxiety/working memory relations are also detectable in children with co-occurring ADHD remains an open empirical question that the current study is well positioned to address.

### Current study

Taken together, previous research paints a mixed picture regarding the association between anxiety and working memory, including evidence for small impairments or small strengths in working memory for children with greater anxiety, or no association between the two. The mixed literature may be accounted for by several proposed mechanisms that are evaluated in the current study, including the multiple components of working memory, multiple dimensions of anxiety, and the high rates of co-occurrence between pediatric anxiety and ADHD.

First, as recommended by Moran (16), bifactor modeling based on eight indicators from two criterion working memory tests (each with 4 distinct memory load conditions) was employed to obtain latent estimates of the domain-general central executive, as well as domain-specific phonological and visuospatial short-term storage systems (e.g., 31, 48). This method was used to address concerns about the limited interpretability of single ‘working memory’ tasks as reflecting specific cognitive processes (16, 34). Second, the current study evaluated the extent to which latent estimates of the physiological arousal and cognitive worry manifestations of anxiety, as well as the variance shared between the two (i.e., common anxiety), have differential associations with each of the three working/short-term memory components. Lastly, given the high co-occurrence between anxiety and ADHD, and the well-documented working memory deficits in children with ADHD (e.g., 31), the current study examined whether any detected working memory/anxiety associations differed between children with versus without ADHD.

We hypothesized that greater common anxiety would be associated with impairments in the domain-general central executive (16). We also expected that higher levels of cognitive worry would be associated with impaired phonological short-term memory and greater physiological arousal symptoms would be associated with worse visuospatial short-term memory (16, 20, 34). No specific hypotheses regarding whether these relations differ for children with versus without ADHD were offered given the paucity of prior research. However, differential associations in children with versus without ADHD seem most likely between anxiety components and central executive working memory given evidence of larger central executive working memory deficits in ADHD compared to short-term memory functioning (31).

## Method

### Participants

The sample included 340 children between the ages of 8 and 13 years (*M* = 10.31, *SD* = 1.39; 144 female participants: **Table 1**) from the Southeastern U.S. recruited through community resources for participation in a clinical research study of the neurocognitive mechanisms underlying pediatric attention and behavior problems. The Florida State University IRB approved the study prior to and throughout data collection, and parents and children gave informed consent/assent. Sample ethnicity consisted of 229 White Non-Hispanic or Latino (67%), 46 Black or African American (13%), 37 multiracial (11%), 23 Hispanic or Latino (7%), and 5 Asian (2%) children. None of the children presented with gross neurological, sensory, or motor impairment; non-stimulant medications that could not be withheld for testing; or history of seizure disorder, psychosis, or intellectual disability.

**Table 1:** Sample and demographic variables.

Variable	ADHD		Non-ADHD		Cohen’s *d*	*p*	Possible Range	Obtained Range		Skewness		Kurtosis	
	(*N*=197)		(*N*=143)										
	*M*	*SD*	*M*	*SD*				ADHD	Non-ADHD	ADHD	Non-ADHD	ADHD	Non-ADHD
Sex (%Male/Female)	63/37		50/50		--	0.02	--	--	--	--	--	--	--
Ethnicity (% B/A/W/H/M)	16/0/69/6/9		10/4/65/8/14		--	0.016	--	--	--	--	--	--	--
Age	10.12	1.39	10.57	1.34	0.33	0.003	8.10-13.50	8.10-13.34	8.29-13.37	0.58	0.37	-0.75	-0.83
SES	47.53	10.16	46.79	12.16	-.07	0.544	11-66	21-66	11-66	-0.5	-0.57	-0.33	-0.03
IQ	101.36	14.9	105.89	12.9	0.32	0.003	73-151	73-138	73-151	0.06	0.06	-0.7	0.64
ADHD Symptoms													
BASC-2/3 Parent ATT T-Score	68.58	6.97	56.71	10.83	-1.35	<.001	10-120	48-86	36-81	-0.4	0.15	0.55	-0.78
BASC-2/3 Parent HYP T-Score	69.45	12.61	56.12	11.49	-1.1	<.001	10-120	42-102	38-91	0	0.92	-0.37	0.83
Anxiety													
Diagnosis (%Yes/No)	34/66		31/69		--	0.623	--	--	--	--	--	--	--
MASC-2 Self-Report Total T-Score	55.84	10.63	54.54	9.66	-0.13	0.249	40-90	40-87	40-81	0.43	0.17	-0.28	-0.74
Working Memory Task Performance													
Phonological Set Size 3	2.62	0.44	2.83	0.24	0.58	<.001	0-3	1.32-3.00	1.83-3.00	-1.2	1.72	0.69	2.71
Phonological Set Size 4	3.19	0.66	3.66	0.34	.85	<.001	0-4	1.17-4.00	2.70-4.00	-0.7	-0.89	-0.05	-0.05
Phonological Set Size 5	3.55	0.92	4.27	0.63	0.9	<.001	0-5	.82-5.00	2.20-5.00	-0.4	-1.23	-0.22	1.1
Phonological Set Size 6	3.13	1.18	3.98	1.15	0.73	<.001	0-6	.33-5.67	.90-6.00	-0.24	-0.55	-0.43	-0.18
Visuospatial Set Size 3	1.92	0.66	2.4	0.48	0.8	<.001	0-3	.17-3.00	.83-3.00	-0.59	-1.07	-0.25	1.09
Visuospatial Set Size 4	2.31	0.91	3.04	0.71	0.87	<.001	0-4	.17-4.00	.83-4.00	-0.25	-0.83	-0.69	0.21
Visuospatial Set Size 5	2.38	1.04	3.21	0.97	0.81	<.001	0-5	.33-4.83	.50-5.00	0.2	-0.4	-0.83	-0.35
Visuospatial Set Size 6	2.13	1.03	3.16	1.14	0.96	<.001	0-6	.33-5.18	.67-5.67	0.81	-0.09	0.48	-0.62

A, Asian; ATT, Attention Problems; B, Black or African American; BASC-2/3, Behavior Assessment Scale for Children, 2^nd^ or 3^rd^ edition; H, Hispanic or Latino; HYP, Hyperactivity; IQ, WISC-V short-form IQ score, fluid reasoning index score, or one-subtest screener; MASC-2, Multidimensional Anxiety Scale for Children, 2^nd^ edition; M, Multiracial; SES, Hollingshead SES total score; W, White Non-Hispanic or Latino. Cohen’s d effect sizes are interpreted as small = .20; medium = .50; large = .80.

### Group assignment

Children and caregivers completed a comprehensive psychoeducational evaluation that included detailed parent semi-structured clinical interviewing using the Kiddie Schedule for Affective Disorders and Schizophrenia for School-Aged Children (K-SADS; 56). The K-SADS (2013 Update) facilitates differential diagnosis according to symptom onset, course, duration, quantity, severity, and impairment in children and adolescents based on DSM-5 criteria (6). Its psychometric properties are well established, including interrater agreement of .93 to 1.00, test-retest reliability of .63 to 1.00, and concurrent (criterion) validity between the K-SADS and psychometrically established parent rating scales (56). This semi-structured clinical interview was supplemented with parent and teacher rating scales from the Behavior Assessment System for Children (BASC-2/3; 57) and ADHD Rating Scale for DSM-IV/5 (ADHD-4/5; 58). Our standard assessment battery also included norm-referenced child internalizing disorder screeners, and additional standardized measures were administered clinically as needed to inform differential diagnosis and accurate assessment of comorbidities (e.g., semi-structured child clinical interviews, additional testing). A psychoeducational report was provided to caregivers; participating children selected a small toy (≤$5) from a prize box.

Children that met all of the following criteria were included in the ADHD group (*n* = 197): (1) DSM-5 diagnosis of ADHD combined (*n* = 132), inattentive (*n* = 57), hyperactive/impulsive (*n* = 6), or other-specified (*n* = 2) presentation by the directing clinical psychologist and multidisciplinary team based on the K-SADS and differential diagnosis considering all available clinical information indicating onset, course, duration, and severity of ADHD symptoms consistent with the ADHD neurodevelopmental syndrome; (2) borderline/clinical elevations on at least one parent and one teacher ADHD subscale (i.e., >90^th^ percentile); and (3) current impairment based on parent-report. Children with any current ADHD presentation specifiers were eligible given the instability of ADHD presentations (e.g., 59). Several children with ADHD also met criteria for common comorbidities based on this comprehensive psychoeducational evaluation, including 67 anxiety disorder (34%), 12 depression (6%), 17 oppositional-defiant disorder (9%)^1^, and 18 autism spectrum disorder (9%). To improve generalizability given that comorbidity is the norm rather than the exception for children with ADHD (60), these children were retained in the sample. Further, 50 children with ADHD (25%) met diagnostic criteria for a learning disorder. 47 children (24%) with ADHD were prescribed psychostimulant medication, which was withheld >24 hours for neurocognitive testing.

The non-ADHD group comprised 143 consecutive case control referrals who did not meet ADHD criteria and included both neurotypical children and children with psychiatric disorders other than ADHD. The non-ADHD group was deliberately recruited to include children who were, and were not, diagnosed with clinical disorders other than ADHD to control for the presence of these diagnoses in the ADHD group. This allows us to draw stronger conclusions about processes implicated in ADHD specifically as opposed to processes that may appear to be impaired in ADHD due to the confounding influence of co-occurring conditions. Thus, participants in this group included neurotypical children (57%) and children with anxiety (31%), depressive (8%), and autism spectrum (10%) disorders. Neurotypical children had normal developmental histories and nonclinical parent/teacher ratings and were recruited through community resources. 10 children without ADHD (7%) met diagnostic criteria for a learning disorder. The ADHD and non-ADHD groups did not differ significantly in the proportion of children with clinical disorders other than ADHD (anxiety, depression, ASD; *p* >.56); however, the ADHD group had higher proportions of ODD and learning disorder as expected (*p*<.001).

96 non-ADHD participants underwent identical evaluations to the ADHD group. Due to funding constraints, the remaining 47 non-ADHD participants (33%) completed abbreviated evaluations that included parent BASC-3 and ADHD-RS-5, a 1 to 2-subtest IQ screener (described below), and detailed developmental, medical, educational, and psychiatric histories. Neurotypical children that received the abbreviated evaluation did not differ from the full evaluation neurotypical subgroup in terms of child-reported anxiety symptoms, age, IQ, SES, and sex (all *p*>.07).

### Procedure

Children completed the working memory tasks as part of a larger battery of neurocognitive testing that involved 1–2 sessions of approximately three hours each. All tasks were counterbalanced to minimize order effects. Children received brief breaks after each task and preset longer breaks every 2–3 tasks to minimize fatigue. For all testing, performance was monitored at all times by the examiner, who was stationed just outside of the testing room (out of the child’s view) to provide a structured setting while minimizing performance improvements associated with examiner demand characteristics (61).

### Measures

#### Socioeconomic status and global intellectual functioning

Hollingshead SES was estimated based on caregiver(s)’ education and occupation (62). In addition, children were administered either a 4-subtest (full evaluation) or a 1–2 subtest (abbreviated battery) Short-Form of the WISC-V (63, 64).

#### Working memory tasks

The Rapport et al. (65) computerized phonological and visuospatial working memory test and administration instructions are identical to those described in Kofler et al. (13). Reliability and validity evidence includes high internal consistency (*α* = .82-.97; 66); 1- to 3-week (*r* = .76-.90; 67) and 10-week (*r* = .73-.84; 30) test-retest reliability; and expected magnitude relations with working memory updating and complex span tasks (*r* = .61-.69; 68). Each working memory test consisted of six trials at each set size (3–6 stimuli/trial), administered in randomized/unpredictable order as recommended (e.g., 69), yielding 24 total trials per task. Five practice trials were administered before each task (80% correct required).

For the *phonological working memory task*, children were presented with a series of jumbled numbers and a capital letter. The letter never appeared in the first or last position of the sequence to minimize potential primacy and recency effects and was counterbalanced across trials to appear an equal number of times in the other serial positions (i.e., position 2, 3, 4, or 5). Children were instructed to verbally recall numbers in order from smallest to largest, and to say the letter last (e.g., 4H62 is correctly recalled as 246H). For the *visuospatial working memory task*, children were shown nine squares arranged in three offset vertical columns. A series of 2.5 cm diameter dots (3, 4, 5, or 6) were presented sequentially in one of the nine squares during each trial, such that no two dots appeared in the same square on a given trial. All dots presented within the squares were black with the exception of one red dot that was counterbalanced across trials to appear an equal number of times in each of the nine squares, but never presented as the first or last stimulus to minimize potential primacy and recency effects. Children reordered the dot locations (black dots in serial order, red dot last) and responded on a modified keyboard. Partial-credit unit scoring (i.e., stimuli correct per trial) was used to index overall working memory performance as recommended (70), computed separately for the phonological and visuospatial working memory tests. Higher scores reflect better working memory.

#### Anxiety symptoms

The *Multidimensional Anxiety Scale for Children* 2nd Edition Self-Report (MASC-2; 71) was completed by children to assess symptoms related to anxiety disorders. Child self-reported anxiety was utilized as our primary indicator of anxiety due to prior work demonstrating that child report of anxiety appears to show greater associations with neurocognitive functions than parent report (54, 72) and appears to be more sensitive to early symptom emergence than parent report (73). The MASC-2 consists of 50 items (4-point Likert scale) and has demonstrated high internal consistency (α=.92) and 1- to 4-week test-retest reliability (*r*=.89; 71). Higher raw scores reflect greater quantity/severity of anxiety symptoms.

Given our goal of assessing two dimensions of anxiety (i.e., cognitive worry and physiological arousal), we examined the MASC-2 item pool to determine if there was a sufficient number of items falling into each subdomain. The 10 items on the Obsessions and Compulsions scale were excluded given that Obsessive-Compulsive and Related Disorders are now classified separately from Anxiety Disorders in the DSM-5 and DSM-5-TR. To this end, the remaining 40 items were judged to fall into one of four categories using an empirically driven rational approach (48, 74). After reviewing definitions of cognitive worry and physiological arousal from the published literature (35–37), the 7 judges (CM, FG, EC, SC, MT, JO, MG) independently determined whether each item reflected (1) cognitive worry, (2) physiological arousal, (3) both, or (4) neither/unclear. Items judged to belong to each category are shown in **Table 2**. Fleiss’ kappa was computed to test the interjudge reliability of our classification of each item into these categories (75) using the R functions fleissm.kappa (from package irr; 76). Fleiss’ kappa for more than two raters (77) indicated substantial agreement between raters, κ = .77, *p* <.001. Internal consistency for the rationally-derived physiological arousal and cognitive worry subdomains was acceptable in the current sample (ω=.81-.84, α=.78-.80).

**Table 2:** Item-level judgments for hypothesized cognitive worry and physiological arousal factor structure.

Paraphrased Item Content	Cognitive Worry/Anxious Apprehension	Physiological Arousal/Anxious Arousal	Both	Neither/Unclear	Inter-judge Agreement (K = 7)	Mean (SD)	Skewness (SE)	Kurtosis (SE)
3. worry about people laughing	X				100%	1.36 (1.13)	0.20 (0.13)	-1.34 (0.26)
4. scared when parents go away	X				86%	1.29 (1.11)	0.26 (0.13)	-1.28 (0.26)
7. going away to camp scares me	X				100%	0.83 (1.05)	0.95 (0.13)	-0.46 (0.26)
10. afraid other kids will make fun	X				100%	1.18 (1.14)	0.44 (0.13)	-1.24 (0.26)
14. getting called on in class	X				100%	0.96 (1.08)	0.68 (0.13)	-0.93 (0.26)
16. afraid people will think I’m stupid	X				100%	0.85 (1.09)	0.93 (0.13)	-0.57 (0.26)
22. worry what people think of me	X				100%	1.11 (1.10)	0.47 (0.13)	-1.16 (0.26)
29. doing something stupid or embarrassing	X				100%	1.32 (1.13)	0.22 (0.13)	-1.34 (0.26)
30. scared riding in car/bus	X				71%	0.37 (0.74)	2.00 (0.13)	3.21 (0.26)
32. nervous to perform in public	X				86%	1.66 (1.21)	-0.21 (0.13)	-1.52 (0.26)
33. scared of bad weather, the dark, heights, animals, or bugs	X				86%	1.26 (1.10)	0.32 (0.13)	-1.23 (0.26)
1. tense/uptight		X			100%	1.09 (0.87)	0.28 (0.13)	-0.80 (0.26)
6. trouble getting breath		X			100%	0.81 (0.93)	0.84 (0.13)	-0.37 (0.26)
8. shaky/jittery		X			100%	1.02 (0.92)	0.47 (0.13)	-0.74 (0.26)
12. dizzy/faint feelings		X			100%	0.80 (0.97)	0.92 (0.13)	-0.31 (0.26)
15. jumpy		X			100%	1.34 (1.14)	0.21 (0.13)	-1.37 (0.26)
18. pains in chest		X			100%	0.82 (0.92)	0.81 (0.13)	-0.37 (0.26)
24. heart races/skips beats		X			100%	0.70 (0.86)	0.99 (0.13)	0.03 (0.26)
27. restless/on edge		X			86%	1.04 (1.08)	0.58 (0.13)	-1.01 (0.26)
31. sick to my stomach		X			100%	1.02 (0.97)	0.48 (0.13)	-0.89 (0.26)
34. hands shake		X			100%	0.83 (0.95)	0.85 (0.13)	-0.36 (0.26)
37. hands feel sweaty/cold		X			100%	1.03 (1.06)	0.60 (0.13)	-0.95 (0.26)
2. asks for permission				X	100%	2.31 (0.84)	-1.10 (0.13)	0.53 (0.26)
5. eyes open for danger				X	71%	1.99 (1.07)	-0.57 (0.13)	-1.05 (0.26)
9. stay near mom/dad				X	100%	2.04 (0.97)	-0.60 (0.13)	-0.77 (0.26)
11. obey parents and teachers				X	100%	2.49 (0.82)	-1.67 (0.13)	2.16 (0.26)
13. check things out first				X	100%	1.78 (1.02)	-0.39 (0.13)	-0.96 (0.26)
17. light on at night				X	100%	1.23 (1.24)	0.37 (0.13)	-1.51 (0.26)
19. avoids going places without family				X	86%	1.39 (1.06)	0.12 (0.13)	-1.20 (0.26)
20. feels strange, weird, or unreal				X	43%	0.67 (0.92)	1.21 (0.13)	0.41 (0.26)
21. do things other people will like				X	100%	1.67 (1.04)	-0.33 (0.13)	-1.04 (0.26)
23. avoids watching scary movies/T.V. shows				X	100%	1.56 (1.20)	-0.11 (0.13)	-1.52 (0.26)
25. stay away from things that upset me				X	71%	2.03 (1.06)	-0.74 (0.13)	-0.75 (0.26)
26. sleep next to someone in family				X	100%	1.20 (1.13)	0.39 (0.13)	-1.26 (0.26)
28. do everything exactly right				X	100%	1.94 (1.01)	-0.65 (0.13)	-0.65 (0.26)
35. make sure things are safe				X	71%	1.91 (1.02)	-0.52 (0.13)	-0.90 (0.26)
36. trouble asking kids to play				X	71%	0.98 (1.15)	0.69 (0.13)	-1.05 (0.26)
38. shy				X	71%	1.39 (1.07)	0.10 (0.13)	-1.23 (0.26)
39. trouble making up mind				X	57%	1.44 (1.11)	0.03 (0.13)	-1.35 (0.26)
40. upset over thought of getting sick			X		43%	0.81 (0.98)	0.94 (0.13)	-0.30 (0.26)
Internal Consistency	ω=.84; α=.80	ω=.81; α=.78	–	ω=.74; α=.71				

Consensus judgments across 7 judges of items from the MASC-2 as reflecting (1) cognitive worry/anxious apprehension, (2) physiological arousal/anxious arousal, (3) both, or (4) neither/unclear. Item content is paraphrased. The 10 items on the Obsessions and Compulsions scale were excluded. Descriptive statistics of each item for the current sample are also depicted.

Descriptively, 11 items each were judged to fall in the cognitive worry and physiological arousal categories, 1 item was judged to fall in both categories, and 17 items were judged to fall in neither of the categories. There was 100% agreement for 26 of the 40 items. Of the remaining items, 6 of 7 judges (86%) agreed for 5 items, and 5 of 7 judges (71%) agreed for 6 items. These minor discrepancies were resolved via consensus by the first and senior authors (CM, MK) based on category definitions derived from prior literature. Finally, there were 3 items with low agreement (**Table 2**), which we therefore classified as Neither/Unclear.

### Bifactor models

Bifactor modeling was used to build latent estimates of the domain-general and domain-specific components of both anxiety and working memory. The current study followed recommendations for bifactor models by Eid et al. (78). As required to properly fit the bifactor models and interpret the general factors, one or more indicators must load onto the general factor but not onto any specific factor (79). These indicators are called ‘reference facets’ or ‘reference domains’ and define the meaning of the general factor (i.e., common anxiety, central executive working memory). The general factors were modeled as uncorrelated with each specific factor, and the specific factors were modeled as uncorrelated with each other, based on the assumption that two distinct sources of variance contribute to an individual’s score on any given item/trial (i.e., variance attributable to the general factor and to a specific factor). This method allows for maximal discrimination between constructs in our bifactor models to provide reliable variance attributable to both domain-general (common anxiety; central executive working memory) and domain-specific (cognitive worry and physiological arousal; phonological and visuospatial short-term memory) processes (78).

#### Anxiety

**Figure 1:**
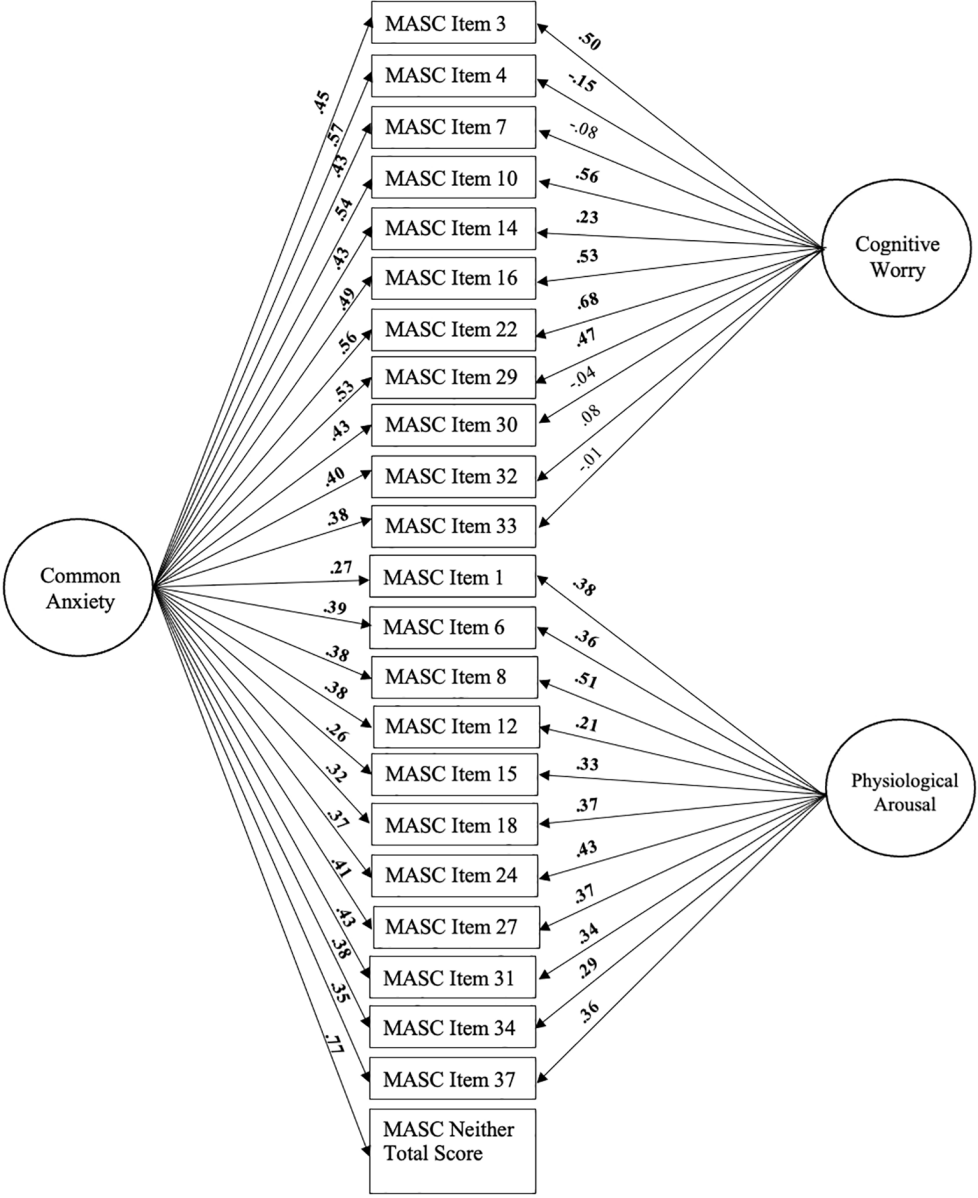
Bifactor-(S-1) model of common anxiety (general factor), and cognitive worry and physiological arousal (specific factors). Standardized loadings are shown. Significant loadings are bolded (all p<.05). Age, sex, and SES are controlled for but not depicted for clarity.

The anxiety bifactor-(*S*-1) model was selected to build latent estimates of domain-general common anxiety and two domain-specific anxiety dimensions (cognitive worry and physiological arousal) based on the evidence reviewed above. To that end, the 22 MASC-2 items described above were modeled to all load onto a general factor (i.e., common anxiety) and a subset of 11 items each loaded onto the specific factors (i.e., cognitive worry, physiological arousal). Additionally, a total score of the 17 MASC-2 items that were judged to be neither cognitive worry or physiological arousal was created and served as the reference facet to define the meaning of the general factor (in this case, common anxiety). See **Figure 1** for a visual depiction of the anxiety bifactor-(*S*-1) model.

#### Working memory

The working memory bifactor-(*S*·I-1) model was selected to build latent estimates of the domain-general central executive working memory and the two domain-specific short-term memory systems (phonological, visuospatial) based on the Baddeley (9) model. In this model, shared variance across working memory tasks with different stimulus modalities (i.e., phonological vs. visuospatial) is attributed to domain-general working memory central executive, whereas unique variance associated with each task is attributed to a domain-specific short-term memory system (i.e., phonological and visuospatial ‘storage/rehearsal’ subsystems; for review, see 31).

**Figure 2:**
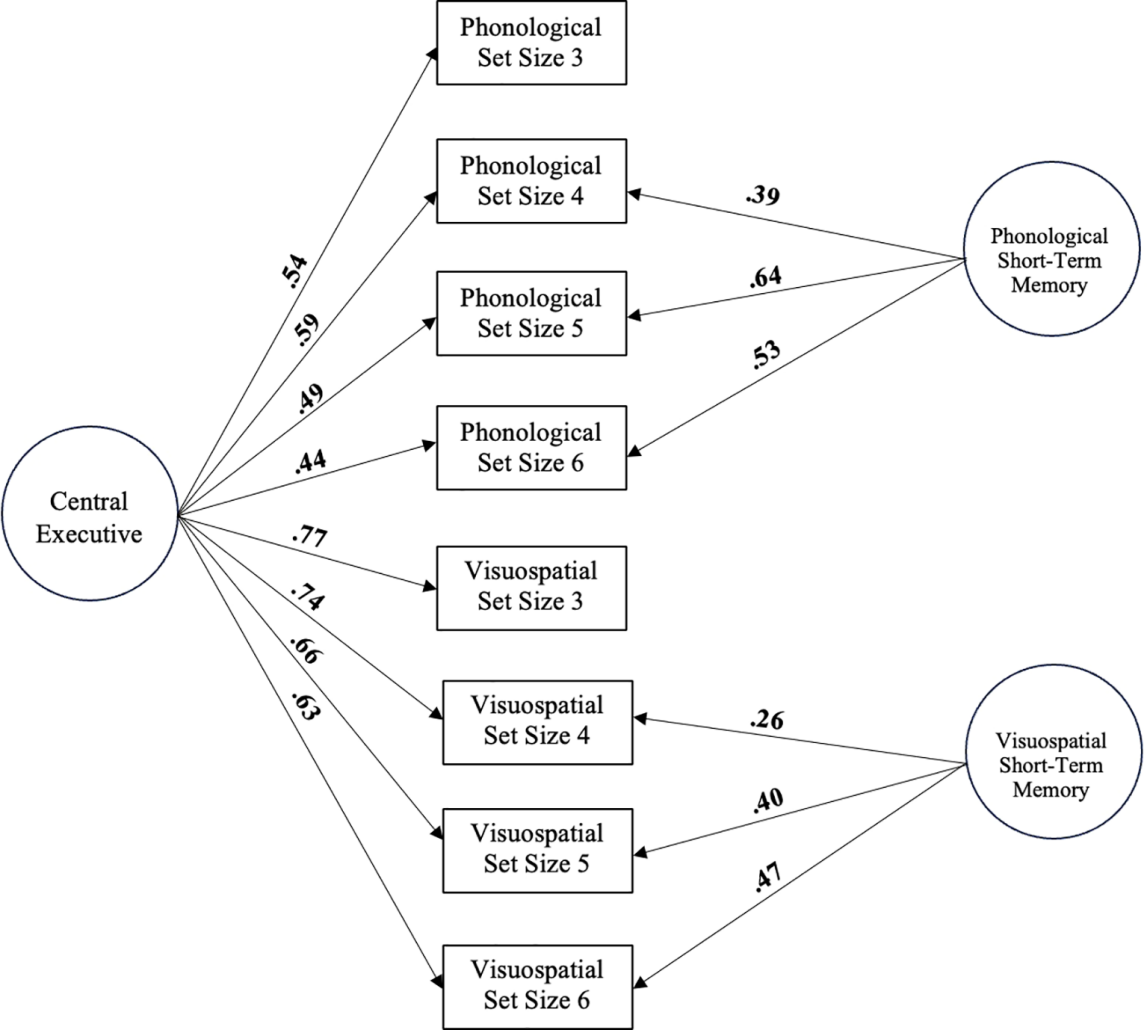
Bifactor-(S·I-1) model of central executive working memory (general factor) and short-term memory (phonological and visuospatial specific factors). Standardized loadings are shown. Significant loadings are bolded (all p<.001). Age, sex, and SES are controlled for but not depicted for clarity.

The working memory bifactor-(*S*·I-1) model used identical procedures as Kofler et al. (31). All 8 indicators (visuospatial and phonological memory set sizes 3, 4, 5, 6) were modeled to load onto the general factor (i.e., central executive working memory) and a subset of indicators were also modeled to load onto a specific short-term memory factor (i.e., phonological or visuospatial). To ensure that the general factor reflected domain-general central executive working memory, we selected 2 reference facets: one phonological and one visuospatial (80). Following Kofler et al. (31), we chose set size 3 from both tasks given that central executive demands remain relatively constant despite increasing set size (9, 49)^2^. See **Figure 2** for a visual depiction of the working memory bifactor-(*S*·I-1) model.

### Transparency and openness

We report how we determined our sample size, all data exclusions (if any), all manipulations, and all study measures. The study was not pre-registered; however, all measure inclusion/exclusion decisions and analytic plans were made *a priori*, prior to accessing the data. A complete correlation matrix is included to allow replication (**Supplementary Table 1**); data/code are available upon reasonable request by emailing the corresponding author. Data were analyzed via structural equation modeling (SEM) using the R package lavaan (81) as implemented in JASP v0.18.3 and R v4.3.3.

### Data analysis overview

Age, sex, and SES were included as covariates in all models. First, the anxiety and working memory bifactor models were built that both included a general factor (common anxiety; central executive working memory), as well as specific factors (cognitive worry and physiological arousal; phonological and visuospatial short-term memory). Model fit was evaluated by comparing these models to 1-factor anxiety and 1-factor working memory models with no specific factors.

Next, we used structural equation modeling to determine if there were differential associations across anxiety dimensions with the three working memory processes. We created a structural model including each anxiety component (i.e., common anxiety, cognitive worry, and physiological arousal) and each working memory component (i.e., central executive, visuospatial and phonological short-term memory) and evaluated correlations between the factors.

Finally, we examined the extent to which any detected relations between anxiety and working memory components differed for children with versus without ADHD using multigroup analysis. In other words, we tested whether the model fit was significantly degraded when the covariances between the anxiety and working/short-term components were constrained to equality across the ADHD and non-ADHD groups by comparing the fit between the constrained and unconstrained models using the chi-square difference test (Δχ2). Lower chi-square values indicate the preferred model (82).

For all confirmatory models, absolute and relative fit were tested. Adequate model fit is indicated by comparative fit index (CFI) and Tucker-Lewis index (TLI) ≥.90, and root mean square error of approximation (RMSEA) ≤.10. For the working memory and anxiety bifactor measurement models, omega total (ω), omega subscale (ωs), explained common variance, and the percentage of uncontaminated correlations were also computed. Omega total (ω) and omega subscale (ωs) index the reliability of the general factor (working memory central executive, common anxiety) and specific factors (phonological and visuospatial short-term memory, cognitive worry and physiological arousal) by providing estimates of the proportion of variance attributable to sources of common and specific variance, respectively; values >.70 are preferred (83). Explained common variance (ECV) indicates the proportion of reliable variance explained by each factor. The percentage of uncontaminated correlations (PUC) is used to assess potential bias from forcing unidimensional data into a multidimensional (bifactor) model. When general factor ECV >.70 and PUC >.70, bias is considered low and the instrument can be interpreted as primarily unidimensional (i.e., the increased complexity of the bifactor structure is likely not warranted; 84). Construct replicability (H) values >.80 suggest a well-defined latent variable that is more likely to be stable across studies (85).

### Power analysis

A series of Monte Carlo simulations were run using the package *simsem* (86) in R (version 4.3.3) to estimate the power of our proposed bifactor models according to the steps outlined in Bader et al. (87). For the proposed working memory bifactor model, we hypothesized general factor loadings (i.e., central executive) of .60 and specific factor loadings (i.e., phonological and visuospatial short-term memory) of .40 based on studies using a subset of the current study’s sample (31, 88). Given these parameters and our sample size of *n* = 340, 5,000 simulations indicated that there was an acceptable convergence rate (99.46%), negligible relative bias (below .03 for all loadings and explained common variance), and very high power to detect significant parameters (above .98 for all loadings).

For the anxiety bifactor model, we hypothesized general factor loadings (i.e., common anxiety) of .60 and specific factor loadings (i.e., cognitive worry and physiological arousal) of .30 based on previous work using similar analyses with child anxiety measures (89–91). Given these parameters and our sample size of *n* = 340, 5,000 simulations indicated that there was an acceptable convergence rate (97.68%), negligible relative bias for all loadings and explained common variance (below .03), and high power to detect significant parameters (above .93 for all loadings).

Power to detect correlations above *r* = .30 between the two bifactors was then estimated using the R package *semPower* (92) given the highly mixed literature regarding anxiety and working memory (22, 28, 52–54). Given the hypothesized bifactor parameters specified above, α-level of .05, and power (1-β) ≥ .80, a sample size of 331 is required to detect correlations above *r* = .30 between the two bifactors. Thus, our sample of *n*=340 is powered to detect clinically relevant associations between components of the working memory and anxiety bifactor models.

## Results

### Preliminary analyses

All raw data were screened for univariate outliers, defined as values three standard deviations above or below the mean for the ADHD and non-ADHD groups separately. Outliers were corrected to the next most extreme value in the sample (0.30% and 0.12% of data points affected for ADHD and non-ADHD groups, respectively). Missing data were imputed using expectation maximization based on all available data and were determined to be missing completely at random (Little’s MCAR test: *χ^2^* = 1014.30, *p* >.99). This affected 0.20% of data points. Sample demographics are shown in **Table 1**. Parent ADHD ratings were significantly higher for the ADHD relative to non-ADHD group as expected. The ADHD and non-ADHD groups did not significantly differ from one another on child report of anxiety symptoms. In contrast, the non-ADHD group was slightly older (*M*=10.57 vs. 10.12; *p*=.003), less likely to be male (*p*=.01), and had slightly higher IQ scores (*M*=105.89 vs. 101.36; *p*=.004), but did not differ from the ADHD group in terms of SES. IQ was not included as a covariate based on compelling statistical, methodological, and conceptual rationale against covarying IQ when investigating cognitive processes in ADHD (93), and because IQ appears to reflect, in part, an outcome rather than a cause of executive function/cognitive control abilities (e.g., 94). In other words, covarying IQ would preclude conclusions regarding executive functioning/cognitive control by fundamentally changing our primary predictor variables, and remove significant variance associated with our predictors and outcomes of interest (93).

### Primary analyses

#### Bifactor measurement models

##### Anxiety bifactor model

First, we created a 1-factor anxiety measurement model in which all 22 cognitive worry and physiological arousal indicators, and the total score variable comprised of items that were classified as falling in neither of these categories, loaded significantly onto the domain general anxiety factor (*β* = .31-.71, all *p* <.001). However, this model did not show adequate fit (**Table 3**). Next, we built the anxiety bifactor (S-1) model by adding the cognitive worry and physiological arousal specific factors to the 1-factor measurement model. This model included the domain-general anxiety (general factor) and the domain-specific cognitive worry and physiological arousal factors (specific factors). As shown in **Figure 1**, all 22 items loaded significantly onto the general factor (all *p* <.001), and all 11 physiological arousal items loaded significantly onto their hypothesized factor (all *p* <.01). The cognitive worry items showed more variability, with four items not significantly loading onto the cognitive worry specific factor (see **Figure 1**). This indicates that these four items (7, 30, 32, 33) do not measure cognitive worry (no true score variance on cognitive worry) after controlling for their association with general anxiety (80). As noted below, study results were unchanged in sensitivity analyses that removed these four items.

**Table 3:** Model fit statistics.

Model	CFI	TLI	RMSEA (90% CI)	SRMR	χ^2^ [df]	Δχ^2^ [df]	ω	ωs	ECV	PUC	H
Bifactor Measurement Models											
Anxiety Single Factor	.71	.68	.07 (.07-.08)	.07	850.20 [296] p <.001	–	–	–	–		–
Anxiety Bifactor	.93	.92	.04 (.03-.05)	.04	393.50 [268] p <.001	456.70 [28] p <.001	.89	.84 (CW) .79 (PA)	.60 (CA) .21 (CW) .19 (PA)	.57	.87 (CA) .71 (CW) .64 (PA)
WM Single Factor	.83	.78	.13 (.11-.14)	.07	263.52 [41] p <.001	–	–	–	–	–	–
WM/STM Bifactor	.95	.90	.08 (.07-.10)	.04	98.59 [29] p <.001	164.93 [12] p <.001	.88	.78 (PH) .82 (VS)	.70 (CE) .19 (PH) .11 (VS)	.68	.85 (CE) .56 (PH) .36 (VS)
Anxiety ➔ WM/STM Model											
	.93	.92	.04 (.03-.04)	.05	696.11 [472] p <.001	–	–	–	–	–	–
ADHD/Non-ADHD Multigroup Models											
Unconstrained	.89	.87	.05 (.04-.05)	.06	1279.36 [944] p <.001	–	–	–	–	–	–
Constrained	.89	.87	.05 (.04-.05)	.06	1289.36 [953] P <.001	10.01 [9] p = .35	–	–	–	–	–

CFI, comparative fit index; ECV, explained common variance; H, construct replicability; PUC, percentage of uncontaminated correlations; RMSEA, root mean square error of approximation; SRMR; STM, short-term memory; TLI, Tucker-Lewis Index; WM, working memory; ω, omega total; ωs, omega subscale; CA, common anxiety; CE, central executive; CW, cognitive worry; PA, physiological arousal; PH, phonological short-term memory; VS, visuospatial short-term memory.

This model showed excellent fit and model fit was significantly improved relative to the 1-factor anxiety measurement model (Δχ2 [28] = 456.70, *p* <.001). The proportion of uncontaminated correlations and explained common variance were both <.70, supporting the multidimensionality of the data (PUC = .57, ECV = .60; 84, 85). Reliability was high for the general factor (ω = .89) and both specific factors (ωs = .79 – .84). Thus, the anxiety bifactor-(*S*-1) model was retained for subsequent analyses. Of note however, construct replicability (H) values for the specific factors were lower than recommended values (**Table 3**), highlighting the importance of additional studies utilizing these methods.

##### WM/STM bifactor model

We then created a 1-factor working memory measurement model in which all 8 indicators loaded significantly onto the domain general working memory factor (*β* = .51-.77, all *p* <.001). However, this model did not show adequate fit (**Table 3**). Next, we built the working/short-term memory bifactor (S·I-1) model by adding the visuospatial and phonological short-term memory specific factors to the 1-factor measurement model. As shown in **Figure 2**, this model included the domain-general central executive (general factor) and the domain-specific phonological short-term memory and visuospatial short-term memory factors (specific factors). This model showed excellent fit, all indicators loaded significantly onto their hypothesized factors (all *p* <.001), and model fit was significantly improved relative to the 1-factor working memory measurement model (Δχ2 [12] = 164.93, *p* <.001). The proportion of uncontaminated correlations and explained common variance were both less than or equal to .70, supporting the multidimensionality of the data (PUC = .68, ECV = .70; Rodriquez et al., 2016; 85). Reliability was high for the general factor (ω = .88) and both specific factors (ωs = .76 – .85). Thus, the working/short-term memory bifactor-(S·I-1) model was retained for subsequent analyses. As with the anxiety bifactor model, construct replicability (H) values for the specific factors were lower than recommended values (**Table 3**) and indicate the need for additional studies measuring and evaluating these constructs.

#### Structural model: associations between anxiety and WM/STM components

**Figure 3:**
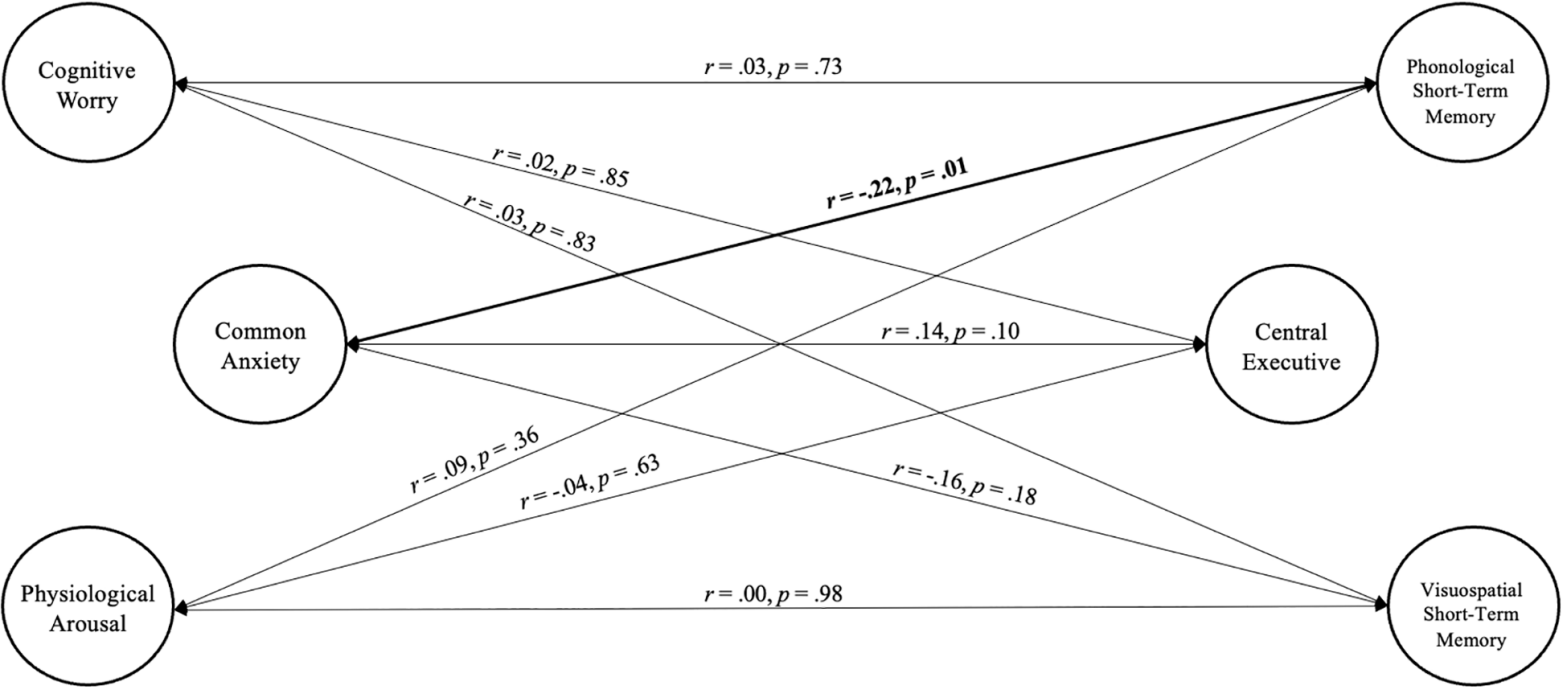
Correlations between bifactor-(S-1) model of common anxiety (general factor), and cognitive worry and physiological arousal (specific factors), and bifactor-(S·I-1) model of central executive working memory (general factor) and short-term memory (phonological and visuospatial specific factors). Age, sex, and SES are controlled for but not depicted for clarity.

We then created the structural model with the three anxiety components and three short-term/working memory components (see **Figure 3**). The model showed excellent fit as shown in **Table 3**. Results indicated that greater common anxiety was significantly associated with worse phonological short-term memory *(r* = -.22, *p* = .01), but not central executive working memory *(r* = .14, *p* = .10) or visuospatial short-term memory (*r* = -.16, *p* = .18). Cognitive worry and physiological arousal were not significantly associated with any of the short-term/working memory components (all *p* >.36).

#### ADHD/Non-ADHD multigroup analysis

Finally, we repeated the model above using a multigroup model (ADHD, Non-ADHD) to test the extent to which the results hold for children with and without ADHD. The unconstrained multigroup model resulted in fit indices slightly below adequate levels (**Table 3**; CFI = .89, TLI = .87, RMSEA = .05). Model fit was similar across the ADHD (CFI = .89, TLI = .87, RMSEA = .05) and non-ADHD groups (CFI = .90, TLI = .88, RMSEA = .05). Constraining the covariances to be equal for both groups did not significantly worsen the overall model fit (Δχ2 [9] = 9.72, *p* = .37), indicating that cognitive worry, physiological arousal, and common anxiety were associated approximately equally with each of the short-term/working memory components for both children with ADHD and without ADHD.

For completeness, we also conducted tests for metric and scalar invariance, which are reported in the **Supplementary Materials** (see **Supplementary Appendix A**). Briefly, there was evidence for scalar invariance, but only partial metric invariance due to minor differences across groups in loadings for specific phonological set sizes that did not impact the pattern or interpretation of results. Comparison of group means on the latent variables indicated that the ADHD group demonstrated worse central executive working memory (*d=*1.03, *p* <.001), as well as phonological and visuospatial short-term memory *(d*=.73, p <.001; *d*=.66, *p* = .002, respectively), abilities relative to the non-ADHD group. The groups did not differ on any of the anxiety factors (all *p* >.30).

### Sensitivity analyses

Overall, our primary findings indicate that cognitive worry and physiological arousal were not associated with any of the working/short-term memory components, but greater common anxiety was associated with worse phonological short-term memory. Next, we conducted sensitivity analyses to probe the extent to which the pattern of results reported above was impacted by our *a priori* decisions to (a) include age, sex, and SES as covariates and (b) retain the four MASC-2 items that failed to load onto the cognitive worry factor. First, we repeated the primary analyses without including age, sex, and SES as covariates. The model continued to demonstrate good fit (CFI = .94, TLI = .93, RMSEA = .04). Greater common anxiety continued to be significantly associated with worse phonological short-term memory (*r* = -.26, *p* = .005) and all other relations remained non-significant. Next, we repeated the primary analyses after removing the four items that failed to load onto the cognitive worry domain (CFI = .94, TLI = .93, RMSEA = .04). Consistent with the primary analyses, greater common anxiety continued to significantly predict worse phonological short-term memory (*r* = -.22, *p* = .01) and all other relations were non-significant.

## Discussion

The current study evaluated several possible explanations for the mixed findings regarding associations between anxiety and working memory in children, including the multi-component nature of working memory, multiple dimensions of anxiety, and high co-occurrence between pediatric anxiety and ADHD. Bifactor modeling was used to establish latent estimates of domain-general (central executive working memory; common anxiety) and domain-specific (phonological and visuospatial short-term memory; cognitive worry and physiological arousal) working/short-term memory and anxiety. Structural models were then used to evaluate relations between the latent factors, with sensitivity analyses probing the impact of our oversampling of children with ADHD, selection of covariates, and decision to retain non-significant items in the anxiety bifactor model. Overall, the current study suggests that greater common anxiety is associated with moderately lower phonological short-term memory (*r* = -.22 to -.26). In contrast – and inconsistent with our hypotheses and theoretical models (16) – cognitive worry and physiological arousal were not uniquely associated with any of the working/short-term memory components.

Interestingly, we found that it is the variance shared between cognitive worry and physiological arousal (i.e., common anxiety) that significantly predicts difficulties in phonological short-term memory. This finding extends prior work that found working memory impairments in children with greater anxiety (22–24) and indicates that the phonological short-term memory storage system may be particularly vulnerable to disruption by anxiety-related thoughts, feelings, and behaviors. That is, greater anxiety may interfere with the temporary storage of phonological information in a variety of ways including competition for neural resources and interference from anxiety-related processes (16), increased dual-processing demands (1, 15), and depleted filtering efficiency (14, 17).

By using bifactor modeling to parse apart the primary components of the working memory/short-term memory system, we were able to address the possibility that prior findings of relations between anxiety and performance on phonological or visuospatial working memory tasks were driven by the central executive rather than either short-term memory system specifically. Our results contrast with the model of working memory and anxiety proposed by Moran (16), in which cognitive worry is related to phonological working memory and physiological arousal is related to visuospatial working memory, whereas domain-general central executive is associated with domain-general common anxiety. Inconsistent with this hypothesis, the latent cognitive worry and physiological arousal factors did not significantly predict any of the latent short-term/working memory components in the current study. Further, our finding that anxiety is related specifically to reduced phonological short-term memory stands in contrast with theoretical models suggesting that the central executive should be most vulnerable to the effects of anxiety (14, 16, 33). That is, we did not find evidence that anxiety is associated with the attentional control processes that are part of the central executive components of working memory (1, 9), but rather the more basic capacity to temporarily store phonological information. The phonological short-term memory system has been found to be more dissociable from central executive working memory processes than the visuospatial system (9, 95), further emphasizing that anxiety may be interfering with processes other than attentional control. Past work has suggested that worry may interfere with phonological storage due to verbal rumination creating dual-processing demands and competition for neural resources (14, 20, 36). While consistent with the current findings, previous studies have been unable to fractionate anxiety and working memory into their component parts. Thus the extent to which prior findings can specifically inform our understanding of cognitive worry and physiological arousal may be limited given their shared variance as demonstrated herein.

These results also contrast with studies suggesting unique associations with each of these anxiety dimensions (20, 34, 39). Interestingly, however, the current findings are consistent with the meta-analytic results from Moran (16), which did not find empirical support for unique predictions from specific domains. This finding was interpreted at the time to be attributable to methodological limitations of the included studies. In light of the current findings, however, it appears likely that specific anxiety subcomponents may not be associated with specific working memory subcomponents – at least for clinically evaluated children. For example, Gustavon and Miyake (39) examined both worry and physiological arousal with separate measures, leaving the possibility that anxiety characteristics shared across these two domains may best account for associations with working memory. Similarly, experimental manipulations of threat-induced anxiety, such as those used by Shackman et al. (34) and Vytal et al. (20) provide a useful paradigm for evaluating causal effects of anxiety in the moment but face similar challenges in parsing apart what is shared versus unique between the anxiety dimensions.

Interpreting what the shared variance between cognitive worry and physiological arousal represents is challenging because most literature focuses on characteristics that distinguish the two anxiety dimensions rather than on their commonality (20, 35, 36, 96, 97). However, some research has proposed temperamental characteristics as cutting across the domains, particularly avoidance temperament or behavioral inhibition (44). These temperaments reflect a tendency toward inhibited behavior that is guided by the possibility of a negative event (98). Consistent with this hypothesis, many of the MASC-2 items judged to reflect neither physiological arousal nor cognitive worry reflected avoidance behaviors and the sum of these items served as the reference facet. As a result, it seems reasonable to conclude that our anxiety general factor reflects avoidance/inhibition, which is a common component of anxiety that is present in both cognitive worry and physiological arousal (44, 99, 100). Research focusing on neural regions that are involved in both cognitive worry and physiological arousal has also provided insight into shared processes between the two dimensions. For example, Castagna and colleagues (42) found that greater cortical thickness in neural regions associated with perceived salience of threat stimuli and cognitive control aspects of emotion regulation was related to high levels of both cognitive worry and physiological arousal. Similarly, a study utilizing event-related potentials as a metric for neural processing found that individuals with elevated physiological arousal and cognitive worry both showed an attentional bias to emotional stimuli (43). These shared neural correlates suggest that heightened processes related to regulation and/or appraisal may be common to both cognitive worry and physiological arousal. Future research will be needed to further characterize domain-general anxiety given our findings that features of anxiety common to both cognitive worry and physiological arousal appear to be implicated in phonological short-term memory processes.

The current study indicated that there were no significant differences in the relations between anxiety and working/short-term memory components for children with versus without ADHD. That is, common anxiety, cognitive worry, and physiological arousal were associated approximately equally with each of the short-term/working memory components for both groups. Thus, our results do not support the hypothesis that anxiety may further impair working memory abilities above and beyond ADHD (21, 22, 51). By the same token, our results were also not in line with the opposite hypothesis that anxiety may buffer against executive function deficits in ADHD through increased effort, greater recruitment of cognitive resources, and/or increased cortical arousal (21, 22, 101–103). Instead, our findings add to this mixed body of literature and suggest that even when anxiety and working memory are fractionated into domain-general and domain-specific components, children with and without ADHD do not exhibit differential associations among these constructs. The mixed literature regarding anxiety and working memory in pediatric ADHD spans a variety of operational definitions of anxiety (e.g, 22, 28, 102, 104) including varied measurement and informant. However, it will be important for future studies to evaluate if the present study results using child self-report of anxiety to fractionate anxiety into multiple components extend to parent report of child anxiety given additional substantive information provided by multiple informants about child psychopathology and high frequency of informant discrepancies (105).

Consistent with prior literature (31, 46), children with ADHD exhibited large magnitude impairments in central executive working memory relative to children without ADHD, whereas visuospatial and phonological short-term memory deficits were larger than expected based on prior literature (12, 31, 47). In contrast, the ADHD and non-ADHD groups did not differ in their levels of cognitive worry, physiological arousal, or domain-general anxiety. However, these results likely reflect, at least in part, our recruitment strategy that emphasized inclusion of clinical controls in addition to typically developing children. Indeed, these results suggest that our recruitment strategy was successful because the two groups did not differ in their anxiety levels, which is consistent with the relatively equal proportion of anxiety diagnoses across groups (approximately one-third of children in each group were diagnosed with an anxiety disorder). Future research with larger samples of neurotypical children as a separate comparison group would provide more clarity regarding the extent to which there may be higher levels of specific dimensions of anxiety in children with ADHD compared to the general population (106). Of course, such an approach would ideally be considered in the context of the limited generalizability of ‘pure’ ADHD groups given that co-occurring conditions are the norm rather than the exception for these children (60).

### Limitations and future directions

The current study has several strengths, including a large, carefully phenotyped sample of children, bifactor modeling to fractionate domain-general and domain-specific components of anxiety and working memory, and the ability to account for the potential impact of the common co-occurrence between ADHD and anxiety. At the same time, the following limitations should be considered when interpreting results. First, the need to fractionate domain general and domain specific factors was highlighted by the poor fit of the single factor working memory and anxiety models, good fit for the bifactor models, and evidence supporting the multidimensionality of both item sets. Further, every latent factor was comprised of at least three significant indicators and showed high reliability. However, the construct replicability (H) values for the specific factors fell below optimal levels, suggesting that future studies may benefit from including additional items when modeling these constructs. Relatedly, our operationalization of cognitive worry and physiological arousal was constrained to the item pool from the MASC-2, which was not developed specifically to measure these dimensions and thus may not have fully captured all aspects of these constructs. Future studies would benefit from developing/utilizing measures designed to specifically differentiate anxiety-related arousal versus worry and/or include a broader sampling of items.

In addition, the current study evaluated trait anxiety, whereas other studies have examined anxiety experienced during the cognitive tasks themselves (i.e., state anxiety; 20, 34). Thus, it is possible that our findings would have differed if we had used an anxiety induction experiment. However, meta-analytic evidence has suggested that relations between anxiety and executive functions do not differ significantly when based on state (induced) versus trait anxiety (16, 25). Nonetheless, future studies may benefit from dual dissociation designs that systematically manipulate working memory demands and state anxiety levels to provide further clarification about the nature and directionality of any detected relations between subcomponents of working memory and anxiety. Studies evaluating both state and trait anxiety in the context of the multicomponent nature of both anxiety and working memory are also needed to determine the mechanisms that may be underlying these relations. For example, Gustavson and Miyake (39) outline two possibilities that may underlie any observed relations between anxiety and working memory: a) the trait effects of anxiety may actually be the result of state anxiety processes elicited during the working memory tasks, or b) cognitive processing may actually differ based on trait-level variability in anxiety.

The current study utilized bifactor modeling to fractionate working memory into component factors across two separate tasks. Central executive working memory, phonological and visuospatial short-term memory, were modeled as uncorrelated to allow for maximal discrimination between each of these constructs (78) due to the study’s aim to examine unique associations between working memory components and multiple dimensions of anxiety. However, given that these distinct processes work in conjunction with one another on any given working/short-term memory task (9), extension of the current findings to additional working memory tasks and performance metrics is needed to further evaluate the robustness of working memory/anxiety relations. We used working memory tasks that assessed reordering processes. In addition to reordering, models of central executive working memory have also highlighted continuous updating and dual-processing (1, 107). Future research utilizing working memory tasks that assess these additional processes are needed to determine if results from the current study extend beyond tasks engaging reordering processes. Additionally, performance on our working memory tests is based on accuracy (i.e., stimuli correct per trial), whereas it is possible that working memory efficiency (i.e., response speeds) may be more vulnerable to the effects of anxiety than working memory accuracy (14, 39) due to the motivational effects of anxiety (108). That is, individuals with high anxiety may put forth increased effort to compensate for reduced working memory capacity, resulting in slower but just as accurate performance (14, 108). Similarly, it is also possible that different levels of anxiety may facilitate rather than impair working memory processing, although this may be unlikely given a recent, relatively large study that tested this hypothesis in a subset of the current sample and did not find support for a curvilinear relation between anxiety and working memory (28).

## Conclusion

Taken together, the current study found that higher levels of domain-general anxiety – but not domain-specific components of anxiety including cognitive worry and physiological arousal – are associated with reduced phonological short-term memory abilities. In contrast, none of the anxiety factors were associated with central executive working memory or visuospatial short-term memory. Given that this was the first study to fractionate both working memory and anxiety into their primary components, these results suggest that prior findings linking anxiety with working memory difficulties may be driven specifically by the interfering effects of anxiety on the temporary storage of phonological information. Interestingly, this pattern was observed equally for children with and without ADHD, suggesting that the findings were not driven by our oversampling for children with ADHD and are not specific to children who have difficulties with working memory as is commonly observed in ADHD samples (12, 13, 31, 46). For practitioners, these findings suggest that the presence of anxiety should be carefully considered when selecting and interpreting neuropsychological testing batteries, as features of anxiety that cut across both cognitive worry and physiological arousal may specifically disrupt short-term memory capacity for verbal information. Similarly, parents and teachers working with children experiencing various forms of anxiety may need to provide visual aids and break down tasks when information is presented verbally, as these children are likely to experience disruptions to their short-term ability to remember this information.

## Data Availability

The data/code will be made available on emailing the corresponding author, without undue reservation.

## Footnotes

1 As recommended in the K-SADS, oppositional-defiant disorder (ODD) was diagnosed only with evidence of multi-informant/multi-setting symptoms.

2 Use of different set size reference facets did not change the pattern and interpretation of results from the bifactor model based on a subset of the current sample (31).

## References

[1] Fosco WD Kofler MJ Groves NB Chan ESM Raiker JS. Which “working” components of working memory aren’t working in youth with ADHD? J Abnormal Child Psychol. (2020) 48:647–60. doi: 10.1007/s10802-020-00621-y PMC7192792 31989344

[2] Sarver DE Rapport MD Kofler MJ Scanlan SW Raiker JS Altro TA. Attention problems, phonological short-term memory, and visuospatial short-term memory: Differential effects on near- and long-term scholastic achievement. Learn Individ Dif. (2012) 22:8–19. doi: 10.1016/j.lindif.2011.09.010

[3] McQuade JD Murray-Close D Shoulberg EK Hoza B. Working memory and social functioning in children. J Exp Child Psychol. (2013) 115:422–35. doi: 10.1016/j.jecp.2013.03.002 23665178

[4] Barkley RA Murphy KR. Impairment in occupational functioning and adult ADHD: The predictive utility of executive function (EF) ratings versus EF tests. Arch Clin Neuropsychol. (2010) 25:157–73. doi: 10.1093/arclin/acq014 PMC2858600 20197297

[5] Huang-Pollock C Shapiro Z Galloway-Long H Weigard A. Is poor working memory a transdiagnostic risk factor for psychopathology? J Abnormal Child Psychol. (2017) 45:1477–90. doi: 10.1007/s10802-016-0219-8 PMC5405018 27783257

[6] American Psychiatric Association. Diagnostic and statistical manual of mental disorders. 5th ed. Washington, DC: American Psychiatric Publishing (2013).

[7] Ouimet AJ Gawronski B Dozois DJA. Cognitive vulnerability to anxiety: A review and an integrative model. Clin Psychol Rev. (2009) 29:459–70. doi: 10.1016/j.cpr.2009.05.004 19552990

[8] Racine N McArthur BA Cooke JE Eirich R Zhu J Madigan S. Global Prevalence of depressive and anxiety symptoms in children and adolescents during COVID-19: A Meta-analysis. JAMA Pediatr. (2021) 175:1142–50. doi: 10.1001/jamapediatrics.2021.2482 PMC8353576 34369987

[9] Baddeley A. Working memory, thought, and action. New York, NY: Oxford University Press (2007). doi: 10.1093/acprof:oso/9780198528012.001.0001

[10] Nitschke JB Heller W Palmieri PA Miller GA. Contrasting patterns of brain activity in anxious apprehension and anxious arousal. Psychophysiology. (1999) 36:628–37. doi: 10.1111/psyp.1999.36.issue-5 10442031

[11] Reimherr FW Marchant BK Gift TE Steans TA. ADHD and anxiety: Clinical significance and treatment implications. Curr Psychiatry Rep. (2017) 19:109. doi: 10.1007/s11920-017-0859-6 29152677

[12] Kasper LJ Alderson RM Hudec KL. Moderators of working memory deficits in children with attention-deficit/hyperactivity disorder (ADHD): A meta-analytic review. Clin Psychol Rev. (2012) 32:605–17. doi: 10.1016/j.cpr.2012.07.001 22917740

[13] Kofler MJ Irwin LN Soto EF Groves NB Harmon SL Sarver DE. Executive functioning heterogeneity in pediatric ADHD. J Abnormal Child Psychol. (2019) 47:273–86. doi: 10.1007/s10802-018-0438-2 PMC6204311 29705926

[14] Eysenck MW Derakshan N Santos R Calvo MG. Anxiety and cognitive performance: Attentional control theory. Emotion. (2007) 7:336–53. doi: 10.1037/1528-3542.7.2.336 17516812

[15] Hirsch CR Mathews A. A cognitive model of pathological worry. Behav Res Ther. (2012) 50:636–46. doi: 10.1016/j.brat.2012.06.007 PMC3444754 22863541

[16] Moran TP. Anxiety and working memory capacity: A meta-analysis and narrative review. psychol Bull. (2016) 142:831–64. doi: 10.1037/bul0000051 26963369

[17] Berggren N. Anxiety and apprehension in visual working memory performance: No change to capacity, but poorer distractor filtering. Anxiety Stress Coping. (2020) 33:299–310. doi: 10.1080/10615806.2020.1736899 32126798

[18] Robinson OJ Vytal K Cornwell BR Grillon C. The impact of anxiety upon cognition: Perspectives from human threat of shock studies. Front Hum Neurosci. (2013) 7:203. doi: 10.3389/fnhum.2013.00203 23730279 PMC3656338

[19] Bredemeier K Berenbaum H. Cross-sectional and longitudinal relations between working memory performance and worry. J Exp Psychopathol. (2013) 4:420–34. doi: 10.5127/jep.032212

[20] Vytal K Cornwell B Arkin N Letkiewicz A Grillon C. The complex interaction between anxiety and cognition: Insight from spatial and verbal working memory. Front Hum Neurosci. (2013) 7:93. doi: 10.3389/fnhum.2013.00093 23542914 PMC3610083

[21] Tannock R. ADHD with anxiety disorders. In T.E. Brown (Ed.) ADHD comorbidities: Handbook for ADHD complications in children and adults. Arlington, VA: American Psychiatric Publishing, Inc (2009) p. 131–55.

[22] Jarrett MA Wolff JC Davis TE Cowart MJ Ollendick TH. Characteristics of children with ADHD and comorbid anxiety. J Attention Disord. (2016) 20:636–44. doi: 10.1177/1087054712452914 22863769

[23] Owens M Stevenson J Hadwin JA Norgate R. Anxiety and depression in academic performance: An exploration of the mediating factors of worry and working memory. School Psychol Int. (2012) 33:433–49. doi: 10.1177/0143034311427433

[24] Visu-Petra L Stanciu O Benga O Miclea M Cheie L. Longitudinal and concurrent links between memory span, anxiety symptoms, and subsequent executive functioning in young children. Front Psychol. (2014) 5:443. doi: 10.3389/fpsyg.2014.00443 24904462 PMC4032945

[25] Shi R Sharpe L Abbott M. A meta-analysis of the relationship between anxiety and attentional control. Clin Psychol Rev. (2019) 72:101754. doi: 10.1016/j.cpr.2019.101754 31306935

[26] Alfonso SV Lonigan CJ. Trait anxiety and adolescent’s academic achievement: The role of executive function. Learn Individ Dif. (2021) 85:101941. doi: 10.1016/j.lindif.2020.101941

[27] Majeed NM Chua YJ Kothari M Kaur M Quek FYX Ng MHS. Anxiety disorders and executive functions: A three-level meta-analysis of reaction time and accuracy. Psychiatry Res Commun. (2023) 3:100100. doi: 10.1016/j.psycom.2022.100100

[28] Marsh CL Harmon SL Cho S Chan ES Gaye F DeGeorge L. Does anxiety systematically bias estimates of executive functioning deficits in pediatric attention-deficit/hyperactivity disorder? Res Child Adolesc Psychopathol. (2024) 52:773–87. doi: 10.1007/s10802-023-01152-y PMC11216413 38157122

[29] Snyder HR Miyake A Hankin BL. Advancing understanding of executive function impairments and psychopathology: Bridging the gap between clinical and cognitive approaches. Front Psychol. (2015) 6:328. doi: 10.3389/fpsyg.2015.00328 25859234 PMC4374537

[30] Kofler MJ Soto EF Singh LJ Harmon SL Jaisle E Smith JN. Executive function deficits in attention-deficit/hyperactivity disorder and autism spectrum disorder. Nature Reviews Psychology. (2024) 3(10):701–19. doi: 10.1038/s44159-024-00350-9 PMC11485171 39429646

[31] Kofler MJ Singh LJ Soto EF Chan ESM Miller CE Harmon SL. Working memory and short-term memory deficits in ADHD: A bifactor modeling approach. Neuropsychology. (2020) 34:686–98. doi: 10.1037/neu0000641 PMC7483636 32437194

[32] Baddeley A Allen RJ Hitch GJ. Investigating the episodic buffer. Psychologica Belgica. (2010) 50:223–43. doi: 10.5334/pb-50-3-4-223

[33] Derakshan N Eysenck MW. Anxiety, processing efficiency, and cognitive performance: New developments from attentional control theory. Eur Psychol. (2009) 14:168–76. doi: 10.1027/1016-9040.14.2.168

[34] Shackman AJ Sarinopoulos I Maxwell JS Pizzagalli DA Lavric A Davidson RJ. Anxiety selectively disrupts visuospatial working memory. Emotion. (2006) 6:40–61. doi: 10.1037/1528-3542.6.1.40 16637749

[35] Nitschke JB Heller W Imig JC McDonald RP Miller GA. Distinguishing dimensions of anxiety and depression. Cogn Ther Res. (2001) 25:1–22. doi: 10.1023/A:1026485530405

[36] Engels AS Heller W Mohanty A Herrington JD Banich MT Webb AG. Specificity of regional brain activity in anxiety types during emotion processing. Psychophysiology. (2007) 44:352–63. doi: 10.1111/j.1469-8986.2007.00518.x 17433094

[37] Heller W Nitschke JB Etienne MA Miller GA. Patterns of regional brain activity differentiate types of anxiety. J Abnormal Psychol. (1997) 106:376–85. doi: 10.1037//0021-843x.106.3.376 9241939

[38] Vytal K Cornwell B Arkin N Grillon C. Describing the interplay between anxiety and cognition: from impaired performance under low cognitive load to reduced anxiety under high load. Psychophysiology. (2012) 49:842–52. doi: 10.1111/j.1469-8986.2012.01358.x PMC3345059 22332819

[39] Gustavson DE Miyake A. Trait worry is associated with difficulties in working memory updating. Cogn Emotion. (2016) 30:1289–303. doi: 10.1080/02699931.2015.1060194 PMC6118341 26208534

[40] Ochsner KN Gross JJ. The cognitive control of emotion. Trends Cogn Sci. (2005) 9:242–9. doi: 10.1016/j.tics.2005.03.010 15866151

[41] Owen AM McMillan KM Laird AR Bullmore E. N-back working memory paradigm: A meta-analysis of normative functional neuroimaging studies. Hum Brain Mapp. (2005) 25:46–59. doi: 10.1002/hbm.20131 15846822 PMC6871745

[42] Castagna PJ Roye S Calamia M Owens-French J Davis TE Greening SG. Parsing the neural correlates of anxious apprehension and anxious arousal in the grey-matter of healthy youth. Brain Imaging Behav. (2018) 12:1084–98. doi: 10.1007/s11682-017-9772-1 28994010

[43] Sass SM Heller W Stewart JL Silton RL Edgar JC Fisher JE. Time course of attentional bias in anxiety: Emotion and gender specificity. Psychophysiology. (2010) 47:247–59. doi: 10.1111/j.1469-8986.2009.00926.x PMC3073148 19863758

[44] Spielberg JM Heller W Silton RL Stewart JL Miller GA. Approach and avoidance profiles distinguish dimensions of anxiety and depression. Cogn Ther Res. (2011) 35:359–71. doi: 10.1007/s10608-011-9364-0

[45] Jarrett MA Ollendick TH. A conceptual review of the comorbidity of attention-deficit/hyperactivity disorder and anxiety: Implications for future research and practice. Clin Psychol Rev. (2008) 28:1266–80. doi: 10.1016/j.cpr.2008.05.004 18571820

[46] Karalunas SL Gustafsson HC Dieckmann NF Tipsord J Mitchell SH Nigg JT. Heterogeneity in development of aspects of working memory predicts longitudinal attention deficit hyperactivity disorder symptom change. J Abnormal Psychol. (2017) 126:774–92. doi: 10.1037/abn0000292 PMC5657320 28782975

[47] Martinussen R Hayden J Hogg-Johnson S Tannock R. A meta-analysis of working memory impairments in children with attention-deficit/hyperactivity disorder. J Am Acad Child Adolesc Psychiatry. (2005) 44:377–84. doi: 10.1097/01.chi.0000153228.72591.73 15782085

[48] Rapport MD Alderson RM Kofler MJ Sarver DE Bolden J Sims V. Working memory deficits in boys with attention-deficit/hyperactivity disorder (ADHD): The contribution of central executive and subsystem processes. J Abnormal Child Psychol. (2008) 36:825–37. doi: 10.1007/s10802-008-9215-y 18317920

[49] Kofler MJ Rapport MD Bolden J Sarver DE Raiker JS. ADHD and working memory: The impact of central executive deficits and exceeding storage/rehearsal capacity on observed inattentive behavior. J Abnormal Child Psychol. (2010) 38:149–61. doi: 10.1007/s10802-009-9357-6 19787447

[50] Raiker JS Rapport MD Kofler MJ Sarver DE. Objectively-measured impulsivity and attention-deficit/hyperactivity disorder (ADHD): Testing competing predictions from the working memory and behavioral inhibition models of ADHD. J Abnormal Child Psychol. (2012) 40:699–713. doi: 10.1007/s10802-011-9607-2 22271141

[51] Schatz DB Rostain AL. ADHD with comorbid anxiety: A review of the current literature. J Attention Disord. (2006) 10:141–9. doi: 10.1177/1087054706286698 17085624

[52] Castagna PJ Calamia M Roye S Greening SG Davis TE. The effects of childhood inattention and anxiety on executive functioning: Inhibition, updating, and shifting. Attention Deficit Hyperactivity Disord. (2019) 11:423–32. doi: 10.1007/s12402-019-00306-7 31089961

[53] Maric M Bexkens A Bögels SM. Is clinical anxiety a risk or a protective factor for executive functioning in youth with ADHD? A Meta-regression analysis. Clin Child Family Psychol Rev. (2018) 21:340–53. doi: 10.1007/s10567-018-0255-8 29484581

[54] Read N Mulraney M McGillivray J Sciberras E. Comorbid anxiety and irritability symptoms and their association with cognitive functioning in children with ADHD. J Abnormal Child Psychol. (2020) 48:1035–46. doi: 10.1007/s10802-020-00658-z 32462307

[55] Rapport MD Orban SA Kofler MJ Friedman LM. Do programs designed to train working memory, other executive functions, and attention benefit children with ADHD? A meta-analytic review of cognitive, academic, and behavioral outcomes. Clin Psychol Rev. (2013) 33:1237–52. doi: 10.1016/j.cpr.2013.08.005 24120258

[56] Kaufman J Birmaher B Brent D Rao U Flynn C Moreci P. Schedule for Affective Disorders and Schizophrenia for School-Age Children-Present and Lifetime Version (K-SADS-PL): Initial reliability and validity data. J Am Acad Child Adolesc Psychiatry. (1997) 36:980–8. doi: 10.1097/00004583-199707000-00021 9204677

[57] Reynolds CR Kamphaus RW. BASC-3: Behavior Assessment System for Children. 3rd ed. Bloomington, MN: Pearson (2015).

[58] DuPaul GJ Power TJ Anastopoulos AD Reid R. ADHD Rating Scale-5 for children and adolescents: Checklists, norms, and clinical interpretation. New York, NY: Guilford Press (2016).

[59] Willcutt EG Nigg JT Pennington BF Solanto MV Rohde LA Tannock R. Validity of DSM-IV attention deficit/hyperactivity disorder symptom dimensions and subtypes. J Abnormal Psychol. (2012) 121:991–1010. doi: 10.1037/a0027347 PMC3622557 22612200

[60] Wilens TE Biederman J Brown S Tanguay S Monuteaux MC Blake C. Psychiatric comorbidity and functioning in clinically referred preschool children and school-age youths with ADHD. J Am Acad Child Adolesc Psychiatry. (2002) 41:262–8. doi: 10.1097/00004583-200203000-00005 11886020

[61] Gomez R Sanson AV. Effects of experimenter and mother presence on attentional performance and activity of hyperactive boys. J Abnormal Child Psychol. (1994) 22:517–29. doi: 10.1007/BF02168935 7822626

[62] Cirino PT Chin CE Sevcik RA Wolf M Lovett M Morris RD. Measuring socioeconomic status: Reliability and preliminary validity for different approaches. Assessment. (2002) 9:145–55. doi: 10.1177/10791102009002005 12066829

[63] Sattler J Dumont R Coalson D. Assessment of children: WISC-V and WPPSI-IV. La Mesa, CA: Sattler Press (2016).

[64] Wechsler D. Wechsler Intelligence Scale for Children. 5th ed. Bloomington, MN: Pearson (2014).

[65] Rapport MD Bolden J Kofler MJ Sarver DE Raiker JS Alderson RM. Hyperactivity in boys with attention-deficit/hyperactivity disorder (ADHD): A ubiquitous core symptom or manifestation of working memory deficits? J Abnormal Child Psychol. (2009) 37:521–34. doi: 10.1007/s10802-008-9287-8 19083090

[66] Kofler MJ Sarver DE Harmon SL Moltisanti A Aduen PA Soto EF. Working memory and organizational skills problems in ADHD. J Child Psychol Psychiatry Allied Disciplines. (2018) 59:57–67. doi: 10.1111/jcpp.12773 PMC5729117 28714075

[67] Sarver DE Rapport MD Kofler MJ Raiker JS Friedman LM. Hyperactivity in attention-deficit/hyperactivity disorder (ADHD): Impairing deficit or compensatory behavior? J Abnormal Child Psychol. (2015) 43:1219–32. doi: 10.1007/s10802-015-0011-1 25863472

[68] Wells EL Kofler MJ Soto EF Schaefer HS Sarver DE. Assessing working memory in children with ADHD: Minor administration and scoring changes may improve digit span backward’s construct validity. Res Dev Disabil. (2018) 72:166–78. doi: 10.1016/j.ridd.2017.10.024 PMC5743590 29156389

[69] Kofler MJ Sarver DE Spiegel JA Day TN Harmon SL Wells EL. Heterogeneity in ADHD: Neurocognitive predictors of peer, family, and academic functioning. Child Neuropsychol. (2017) 23:733–59. doi: 10.1080/09297049.2016.1205010 PMC6083022 27472007

[70] Conway ARA Kane MJ Bunting MF Hambrick DZ Wilhelm O Engle RW. Working memory span tasks: A methodological review and user’s guide. Psychonomic Bull Rev. (2005) 12:769–86. doi: 10.3758/BF03196772 16523997

[71] March J. Manual for the Multidimensional Anxiety Scale for Children. 2nd ed. Toronto, Ontario, Canada: Multi-Health Systems (2013).

[72] Bloemsma JM Boer F Arnold R Banaschewski T Faraone SV Buitelaar JK. Comorbid anxiety and neurocognitive dysfunctions in children with ADHD. Eur Child Adolesc Psychiatry. (2013) 22:225–34. doi: 10.1007/s00787-012-0339-9 23086381

[73] Cole DA Tram JM Martin JM Hoffman KB Ruiz MD Jacquez FM. Individual differences in the emergence of depressive symptoms in children and adolescents: A longitudinal investigation of parent and child reports. J Abnormal Psychol. (2002) 111:156–65. doi: 10.1037/0021-843X.111.1.156 11866168

[74] Clark LA Watson D. Constructing validity: Basic issues in objective scale development. psychol Assess. (1995) 7:309–19. doi: 10.1037/1040-3590.7.3.309

[75] Nunnally JC Bernstein IH. Psychological theory. New York, NY: MacGraw-Hill (1994).

[76] Gamer M Lemon J Fellows I Singh P. *Irr: various coefficients of interrater reliability and agreement* (R package version 0.84.1). (2019).

[77] Siegel S Castellan NJ. Nonparametric statistics for behavioral sciences. 2nd ed. New York, NY: McGraw-Hill (1988).

[78] Eid M Geiser C Koch T Heene M. Anomalous results in G-factor models: Explanations and alternatives. psychol Methods. (2017) 22:541–62. doi: 10.1037/met0000083 27732052

[79] Eid M Krumm S Koch T Schulze J. Bifactor models for predicting criteria by general and specific factors: Problems of nonidentifiability and alternative solutions. J Intell. (2018) 6:42. doi: 10.3390/jintelligence6030042 31162469 PMC6480823

[80] Heinrich M Geiser C Zagorscak P Burns GL Bohn J Becker SP. On the meaning of the “P factor” in symmetrical bifactor models of psychopathology: Recommendations for future research from the bifactor-(S-1) perspective. Assessment. (2023) 30:487–507. doi: 10.1177/10731911211060298 34861784 PMC9999288

[81] Rosseel Y. Lavaan: An R package for structural equation modeling and more. Version 0.5–12. J Stat Softw. (2012) 48:1–36. doi: 10.18637/jss.v048.i02

[82] Satorra A Bentler PM. Ensuring positiveness of the scaled difference chi-square test statistic. Psychometrika. (2010) 75:243–8. doi: 10.1007/s11336-009-9135-y PMC2905175 20640194

[83] Rodriguez A Reise SP Haviland MG. Evaluating bifactor models: Calculating and interpreting statistical indices. psychol Methods. (2016) 21:137–50. doi: 10.1037/met0000045 26523435

[84] Rodriguez A Reise SP Haviland MG. Applying bifactor statistical indices in the evaluation of psychological measures. J Pers Assess. (2016) 98:223–37. doi: 10.1080/00223891.2015.1089249 26514921

[85] Watkins MW. The reliability of multidimensional neuropsychological measures: From alpha to omega. Clin Neuropsychologist. (2017) 31:1113–26. doi: 10.1080/13854046.2017.1317364 28429633

[86] Pornprasertmanit S Miller P Schoemann A Jorgensen T Quick C. *Package simsem: SIMulated structural equation modeling*(0.5-16.908). (2021).

[87] Bader M Jobst LJ Moshagen M. Sample size requirements for bifactor models. Struct Equation Modeling. (2022) 29:772–83. doi: 10.1080/10705511.2021.2019587

[88] Gaye F Groves NB Chan ESM Cole AM Jaisle EM Soto EF. Working memory and math skills in children with and without ADHD. Neuropsychology. (2024) 38:1–16. doi: 10.1037/neu0000920 37917437 PMC10842998

[89] DeSousa DA Zibetti MR Trentini CM Koller SH Manfro GG Salum GA. Screen for child anxiety related emotional disorders: Are subscale scores reliable? A bifactor model analysis. J Anxiety Disord. (2014) 28:966–70. doi: 10.1016/j.janxdis.2014.10.002 25445087

[90] Ebesutani C Reise SP Chorpita BF Ale C Regan J Young J. The Revised Child Anxiety and Depression Scale-Short Version: Scale reduction via exploratory bifactor modeling of the broad anxiety factor. psychol Assess. (2012) 24:833–45. doi: 10.1037/a0027283 22329531

[91] Klaufus L Verlinden E van der Wal M Kösters M Cuijpers P Chinapaw M. Psychometric evaluation of two short versions of the Revised Child Anxiety and Depression Scale. BMC Psychiatry. (2020) 20:47. doi: 10.1186/s12888-020-2444-5 32024481 PMC7003441

[92] Moshagen M Erdfelder E. A new strategy for testing structural equation models. Struct Equation Modeling: A Multidiscip J. (2016) 23:54–60. doi: 10.1080/10705511.2014.950896

[93] Dennis M Francis DJ Cirino PT Schachar R Barnes MA Fletcher JM. Why IQ is not a covariate in cognitive studies of neurodevelopmental disorders. J Int Neuropsychol Society: JINS. (2009) 15:331–43. doi: 10.1017/S1355617709090481 PMC3075072 19402919

[94] Engle RW Tuholski SW Laughlin JE Conway ARA. Working memory, short-term memory, and general fluid intelligence: A latent-variable approach. J Exp Psychol. (1999) 128:309–31. doi: 10.1037/0096-3445.128.3.309 10513398

[95] Kane MJ Hambrick DZ Tuholski SW Wilhelm O Payne TW Engle RW. The generality of working memory capacity: a latent-variable approach to verbal and visuospatial memory span and reasoning. Journal of Experimental Psychology (2004) 133(2):189–217. doi: 10.1037/0096-3445.133.2.189 15149250

[96] Craske MG Rauch SL Ursano R Prenoveau J Pine DS Zinbarg RE. What is an anxiety disorder? Depression and Anxiety. (2009) 26(12):1066–85. doi: 10.1002/da.20633 19957279

[97] Sharp PB Miller GA Heller W. Transdiagnostic dimensions of anxiety: Neural mechanisms, executive functions, and new directions. International Journal of Psychophysiology. 2015 98(2 Pt 2):365–77. doi: 10.1016/j.ijpsycho.2015.07.001 26156938

[98] Elliot AJ Thrash TM. Approach-avoidance motivation in personality: Approach and avoidance temperaments and goals. J Pers Soc Psychol. (2002) 82:804–18. doi: 10.1037/0022-3514.82.5.804 12003479

[99] Beesdo-Baum K Knappe S. Developmental epidemiology of anxiety disorders. Child and Adolescent Psychiatric Clinics of North America. (2012) 21(3):457–78. doi: 10.1016/j.chc.2012.05.001 22800989

[100] Lebowitz ER Shic F Campbell D Basile K Silverman WK. Anxiety sensitivity moderates behavioral avoidance in anxious youth. Behaviour Research and Therapy. (2015) 74:11–7. doi: 10.1016/j.brat.2015.08.009 PMC4895193 26348546

[101] Arnsten AFT. Toward a new understanding of attention-deficit hyperactivity disorder pathophysiology: An important role for prefrontal cortex dysfunction. CNS Drugs. (2009) 23:33–41. doi: 10.2165/00023210-200923000-00005 19621976

[102] Ruf BM Bessette KL Pearlson GD Stevens MC. Effect of trait anxiety on cognitive test performance in adolescents with and without attention-deficit/hyperactivity disorder. J Clin Exp Neuropsychol. (2017) 39:434–48. doi: 10.1080/13803395.2016.1232373 27690740

[103] Shaw P Eckstrand K Sharp W Blumenthal J Lerch JP Greenstein D. Attention-deficit/hyperactivity disorder is characterized by a delay in cortical maturation. Proc Natl Acad Sci. (2007) 104:19649–54. doi: 10.1073/pnas.0707741104 PMC2148343 18024590

[104] Vance A Ferrin M Winther J Gomez R. Examination of spatial working memory performance in children and adolescents with attention deficit hyperactivity disorder, combined type (ADHD-CT) and anxiety. J Abnormal Child Psychol. (2013) 41:891–900. doi: 10.1007/s10802-013-9721-4 23378043

[105] De Los Reyes A. Introduction to the special section: More than measurement error: Discovering meaning behind informant discrepanciesin clinical assessments of children and adolescents. J Cllnical Child Adolesc Psychol. (2011) 40:1–9. doi: 10.1080/15374416.2011.533405 21229439

[106] Larson K Russ SA Kahn RS Halfon N. Patterns of comorbidity, functioning, and service use for US children with ADH. Pediatrics. (2011) 127:462–70. doi: 10.1542/peds.2010-0165 PMC3065146 21300675

[107] Wager TD Smith EE. Neuroimaging studies of working memory. Cognitive Affective Behav Neurosci. (2003) 3:255–74. doi: 10.3758/CABN.3.4.255 15040547

[108] Visu-Petra L Miclea M Visu-Petra G. Individual differences in anxiety and executive functioning: A multidimensional view. Int J Psychol. (2013) 48:649–59. doi: 10.1080/00207594.2012.656132 22348375

