# Supplementary material for: Associations between anxiety and working memory components in clinically evaluated children with and without ADHD
Source: Front Psychiatry. 2025 Jun 13;16:1536942. doi: 10.3389/fpsyt.2025.1536942 (PMC12202433; doi:10.3389/fpsyt.2025.1536942)
Supplement: Supplementary file 1 [file Supplementaryfile1.docx]

**Supplementary Table 1.** *Zero-order correlations*

|  | 1 | 2 | 3 | 4 | 5 | 6 | 7 | 8 | 9 | 10 | 11 | 12 | 13 | 14 | 15 | 16 | 17 | 18 | 19 | 20 | 21 | 22 | 23 | 24 | 25 | 26 | 27 | 28 | 29 | 30 | 31 | 32 | 33 | 34 | 35 |
| --- | --- | --- | --- | --- | --- | --- | --- | --- | --- | --- | --- | --- | --- | --- | --- | --- | --- | --- | --- | --- | --- | --- | --- | --- | --- | --- | --- | --- | --- | --- | --- | --- | --- | --- | --- |
| 1 Age | 1 |  |  |  |  |  |  |  |  |  |  |  |  |  |  |  |  |  |  |  |  |  |  |  |  |  |  |  |  |  |  |  |  |  |  |
| 2 Sex | -.03 | 1 |  |  |  |  |  |  |  |  |  |  |  |  |  |  |  |  |  |  |  |  |  |  |  |  |  |  |  |  |  |  |  |  |  |
| 3 SES | .04 | .02 | 1 |  |  |  |  |  |  |  |  |  |  |  |  |  |  |  |  |  |  |  |  |  |  |  |  |  |  |  |  |  |  |  |  |
| 4 ADHD | ***-.16*** | **-.13** | .03 | 1 |  |  |  |  |  |  |  |  |  |  |  |  |  |  |  |  |  |  |  |  |  |  |  |  |  |  |  |  |  |  |  |
| 5 SFIQ | -.04 | .08 | ***.30*** | ***-.16*** | 1 |  |  |  |  |  |  |  |  |  |  |  |  |  |  |  |  |  |  |  |  |  |  |  |  |  |  |  |  |  |  |
| 6 MASC_3 | .05 | .02 | .05 | .02 | .09 | 1 |  |  |  |  |  |  |  |  |  |  |  |  |  |  |  |  |  |  |  |  |  |  |  |  |  |  |  |  |  |
| 7 MASC_4 | ***-.21*** | .06 | ***-.14*** | .04 | -.07 | ***.24*** | 1 |  |  |  |  |  |  |  |  |  |  |  |  |  |  |  |  |  |  |  |  |  |  |  |  |  |  |  |  |
| 8 MASC_7 | ***-.11*** | .00 | -.02 | .04 | .03 | ***.14*** | ***.31*** | 1 |  |  |  |  |  |  |  |  |  |  |  |  |  |  |  |  |  |  |  |  |  |  |  |  |  |  |  |
| 9 MASC_10 | -.03 | .04 | .06 | .07 | .07 | ***.55*** | ***.25*** | ***.22*** | 1 |  |  |  |  |  |  |  |  |  |  |  |  |  |  |  |  |  |  |  |  |  |  |  |  |  |  |
| 10 MASC_14 | .05 | -.00 | -.00 | .05 | -.06 | ***.26*** | ***.21*** | ***.18*** | ***.37*** | 1 |  |  |  |  |  |  |  |  |  |  |  |  |  |  |  |  |  |  |  |  |  |  |  |  |  |
| 11 MASC_16 | .07 | .01 | .09 | .09 | .09 | ***.40*** | ***.18*** | ***.19*** | ***.57*** | ***.37*** | 1 |  |  |  |  |  |  |  |  |  |  |  |  |  |  |  |  |  |  |  |  |  |  |  |  |
| 12 MASC_22 | .05 | .03 | .10 | .00 | **.11** | ***.57*** | ***.20*** | ***.16*** | ***.64*** | ***.35*** | ***.60*** | 1 |  |  |  |  |  |  |  |  |  |  |  |  |  |  |  |  |  |  |  |  |  |  |  |
| 13 MASC_29 | ***.15*** | -.04 | .03 | .03 | ***.17*** | ***.45*** | ***.19*** | ***.14*** | ***.47*** | ***.31*** | ***.50*** | ***.60*** | 1 |  |  |  |  |  |  |  |  |  |  |  |  |  |  |  |  |  |  |  |  |  |  |
| 14 MASC_30 | -.01 | .07 | .01 | .08 | -.02 | ***.15*** | ***.26*** | ***.25*** | ***.16*** | ***.17*** | ***.20*** | ***.21*** | ***.21*** | 1 |  |  |  |  |  |  |  |  |  |  |  |  |  |  |  |  |  |  |  |  |  |
| 15 MASC_32 | .03 | -.01 | -.07 | .08 | .06 | ***.20*** | ***.16*** | **.11** | ***.22*** | ***.28*** | ***.18*** | ***.27*** | ***.25*** | ***.18*** | 1 |  |  |  |  |  |  |  |  |  |  |  |  |  |  |  |  |  |  |  |  |
| 16 MASC_33 | .02 | .02 | .04 | -.02 | ***.19*** | **.12** | .10 | ***.15*** | **.12** | ***.18*** | ***.18*** | ***.21*** | ***.22*** | ***.17*** | ***.22*** | 1 |  |  |  |  |  |  |  |  |  |  |  |  |  |  |  |  |  |  |  |
| 17 MASC_1 | .01 | -.06 | **.13** | -.09 | ***.21*** | ***.18*** | .10 | **.13** | **.12** | **.11** | ***.20*** | ***.19*** | ***.22*** | **.12** | .08 | **.13** | 1 |  |  |  |  |  |  |  |  |  |  |  |  |  |  |  |  |  |  |
| 18 MASC_6 | .01 | -.03 | .04 | -.02 | .02 | **.11** | ***.19*** | ***.16*** | .10 | ***.17*** | ***.16*** | ***.19*** | ***.16*** | ***.24*** | ***.21*** | ***.22*** | ***.26*** | 1 |  |  |  |  |  |  |  |  |  |  |  |  |  |  |  |  |  |
| 19 MASC_8 | .07 | -.09 | -.01 | .02 | .07 | ***.19*** | ***.21*** | ***.18*** | ***.16*** | ***.15*** | **.12** | ***.17*** | ***.22*** | ***.23*** | ***.27*** | ***.16*** | ***.33*** | ***.28*** | 1 |  |  |  |  |  |  |  |  |  |  |  |  |  |  |  |  |
| 20 MASC_12 | -.07 | .13 | .05 | -.01 | .07 | ***.18*** | ***.22*** | ***.17*** | ***.18*** | **.13** | ***.22*** | ***.16*** | ***.18*** | ***.17*** | **.13** | ***.14*** | ***.19*** | ***.20*** | ***.22*** | 1 |  |  |  |  |  |  |  |  |  |  |  |  |  |  |  |
| 21 MASC_15 | .07 | -.00 | .02 | .05 | **.11** | **.14** | **.11** | .07 | .06 | ***.17*** | **.12** | ***.15*** | ***.15*** | .09 | ***.31*** | ***.16*** | ***.19*** | ***.16*** | ***.38*** | **.12** | 1 |  |  |  |  |  |  |  |  |  |  |  |  |  |  |
| 22 MASC_18 | -.06 | -.00 | -.01 | .08 | .08 | .09 | ***.16*** | **.14** | .10 | ***.16*** | **.14** | ***.17*** | ***.15*** | **.11** | .06 | .09 | ***.23*** | ***.31*** | ***.24*** | ***.31*** | .10 | 1 |  |  |  |  |  |  |  |  |  |  |  |  |  |
| 23 MASC_24 | .04 | -.03 | -.01 | .08 | .02 | ***.14*** | ***.20*** | ***.16*** | ***.17*** | **.11** | ***.23*** | ***.20*** | ***.24*** | ***.17*** | ***.16*** | .07 | ***.16*** | ***.35*** | ***.35*** | ***.21*** | ***.19*** | ***.21*** | 1 |  |  |  |  |  |  |  |  |  |  |  |  |
| 24 MASC_27 | -.05 | .01 | .07 | **.12** | **.12** | ***.19*** | ***.25*** | **.12** | ***.16*** | ***.17*** | ***.28*** | ***.23*** | ***.22*** | ***.19*** | ***.18*** | ***.19*** | ***.25*** | ***.27*** | ***.33*** | ***.25*** | ***.34*** | ***.32*** | ***.33*** | 1 |  |  |  |  |  |  |  |  |  |  |  |
| 25 MASC_31 | -.04 | .02 | -.01 | .08 | .04 | **.12** | ***.19*** | ***.15*** | ***.23*** | **.13** | ***.17*** | ***.21*** | ***.24*** | ***.25*** | **.13** | ***.18*** | ***.21*** | ***.27*** | ***.29*** | ***.25*** | ***.27*** | ***.29*** | ***.29*** | ***.24*** | 1 |  |  |  |  |  |  |  |  |  |  |
| 26 MASC_34 | -.01 | -.02 | -.06 | .08 | .06 | **.20** | ***.15*** | ***.18*** | ***.25*** | **.11** | ***.27*** | ***.28*** | ***.24*** | ***.23*** | ***.22*** | ***.18*** | ***.19*** | ***.24*** | ***.29*** | ***.19*** | .10 | ***.23*** | ***.33*** | ***.18*** | ***.28*** | 1 |  |  |  |  |  |  |  |  |  |
| 27 MASC_37 | .01 | .00 | -.05 | -.02 | -.04 | **.11** | .10 | .10 | ***.20*** | ***.19*** | ***.16*** | **.14** | ***.19*** | ***.15*** | ***.14*** | .10 | ***.19*** | ***.23*** | ***.30*** | ***.17*** | **.12** | ***.32*** | ***.30*** | ***.19*** | ***.35*** | ***.30*** | 1 |  |  |  |  |  |  |  |  |
| 28 Neither | **-.12** | .01 | -.01 | .02 | ***.15*** | ***.33*** | ***.45*** | ***.31*** | ***.40*** | ***.32*** | ***.34*** | ***.41*** | ***.43*** | ***.28*** | ***.32*** | ***.36*** | ***.19*** | ***.29*** | ***.26*** | ***.29*** | ***.20*** | ***.24*** | ***.26*** | ***.29*** | ***.34*** | ***.24*** | ***.32*** | 1 |  |  |  |  |  |  |  |
| 29 PH3 | ***.20*** | *.20* | .04 | ***-.28*** | ***.26*** | .04 | .02 | -.02 | .05 | -.07 | -.02 | .04 | .09 | -.10 | -.00 | **.12** | .08 | .07 | .05 | .03 | .06 | -.05 | .02 | -.06 | .08 | .09 | .02 | .08 | 1 |  |  |  |  |  |  |
| 30 PH4 | ***.33*** | .05 | .04 | ***-.39*** | ***.26*** | .09 | -.08 | -.09 | .00 | -.07 | -.04 | .09 | **.13** | -.10 | .02 | .07 | **.12** | .05 | .07 | -.04 | .04 | .01 | .04 | -.09 | .04 | .02 | .06 | .04 | ***.52*** | 1 |  |  |  |  |  |
| 31 PH5 | ***.37*** | .01 | ***.14*** | ***-.41*** | ***.30*** | .04 | -.10 | -.10 | -.04 | -.01 | -.05 | .03 | .06 | ***-.15*** | .01 | .07 | **.11** | .03 | .00 | -.09 | .03 | -.06 | .00 | -.10 | .01 | -.01 | .02 | -.01 | ***.47*** | ***.61*** | 1 |  |  |  |  |
| 32 PH6 | ***.31*** | -.09 | ***.20*** | ***-.34*** | ***.25*** | .02 | *-.19* | -.09 | -.04 | -.04 | -.03 | -.00 | .03 | -.08 | .00 | .05 | .07 | -.05 | .03 | -.05 | .03 | -.06 | -.06 | ***-.15*** | -.04 | -.08 | -.02 | **-.11** | ***.31*** | ***.52*** | ***.61*** | 1 |  |  |  |
| 33 VS3 | ***.27*** | -.01 | .10 | ***-.37*** | ***.24*** | .07 | -.05 | -.03 | .06 | .05 | -.00 | .06 | ***.17*** | -.04 | .07 | *.14* | .08 | .07 | .05 | -.01 | .00 | -.04 | **-.13** | -.06 | .05 | .03 | .01 | .05 | ***.34*** | ***.47*** | ***.40*** | ***.39*** | 1 |  |  |
| 34 VS4 | ***.31*** | -.00 | **.11** | ***-.40*** | ***.30*** | .08 | -.08 | -.03 | .05 | .02 | .01 | .07 | ***.14*** | -.06 | .05 | **.13** | **.13** | .06 | .04 | -.02 | .03 | -.05 | -.07 | -.03 | .01 | -.03 | -.03 | -.01 | ***.35*** | ***.45*** | ***.39*** | ***.37*** | ***.64*** | 1 |  |
| 35 VS5 | ***.36*** | -.04 | **.13** | ***-.37*** | ***.30*** | .07 | -.09 | -.07 | .06 | .03 | .05 | .08 | **.11** | -.02 | **.11** | .08 | **.13** | .05 | .06 | -.01 | .07 | .03 | .01 | .03 | .01 | -.01 | .05 | .06 | ***.31*** | ***.41*** | ***.43*** | ***.41*** | ***.57*** | ***.66*** | 1 |
| 36 VS6 | ***.38*** | -.08 | **.16** | ***-.43*** | ***.23*** | .06 | *-.12* | -.08 | -.03 | -.05 | .00 | .05 | .08 | -.05 | .00 | .08 | .05 | .00 | -.02 | .01 | -.05 | -.05 | -.06 | -.10 | -.01 | .06 | .02 | -.03 | ***.34*** | ***.44*** | ***.45*** | ***.43*** | ***.54*** | ***.64*** | ***.66*** |

*Note*. Correlation coefficients in bold are statistically significant at the .05 level. Correlation coefficients in italics are statistically significant at the .01 level. SFIQ = Wechsler Intelligence Scale for Children Fifth Edition Short-Form Intelligence Quotient; 6 – 27 = individual items on the Multidimensional Anxiety Scale for Children Second Edition; Neither = Neither/Unclear scale on the MASC; 29 – 32 = Phonological Working Memory Set Sizes 3, 4, 5, and 6; 33 – 36 = Visuospatial Working Memory Set Sizes 3, 4, 5, and 6.

**Supplementary Appendix A**

**Metric and Scalar Invariance Testing**

Metric non-invariance was found in the working memory prediction model across ADHD and non-ADHD groups. Constraining the factor loadings to be equal for both groups significantly worsened model fit (Δχ2 [53] = 89.34, *p* = .001). Further examination of the impact of constraining specific parameters indicated that the loading of phonological working memory set size 4 on the general working memory factor and phonological working memory set size 6 on the specific phonological short-term memory factor had the greatest contribution to model fit. Phonological working memory set size 4 contributed to the general working memory factor to a greater degree in the ADHD group (β = .56, *p* < .001) compared to the non-ADHD group (β = .20, *p* = .10). In contrast, phonological working memory set size 6 contributed to the specific phonological short-term memory factor to a greater degree in the non-ADHD group (β = .68, *p* < .001) compared to the ADHD group (β = .54, *p* < .001). Freeing these parameters resulted in partial metric invariance across the ADHD and non-ADHD group (Δχ2 [51] = 63.91, *p* = .11). Constraining the covariances to be equal for both groups did not significantly worsen model fit compared to the partial metric invariance model (Δχ2 [9] = 7.19, *p* = .62), indicating that cognitive worry, physiological arousal, and common anxiety were associated approximately equally to each of the short-term/working memory components for both children with ADHD and without ADHD. Scalar invariance was supported in the working memory prediction model across ADHD and non-ADHD groups when allowing the two previously mentioned parameters to remain free. Constraining the intercepts to be equal for both groups did not significantly worsen model fit compared to the partial metric invariance model (Δχ2 [25] = 30.41, *p* = .21)
